# Drought Sensitivity of the Carbon Isotope Composition of Leaf Dark-Respired CO_2_ in C_3_ (*Leymus chinensis*) and C_4_ (*Chloris virgata* and *Hemarthria altissima*) Grasses in Northeast China

**DOI:** 10.3389/fpls.2017.01996

**Published:** 2017-12-05

**Authors:** Shangzhi Zhong, Hua Chai, Yueqiao Xu, Yan Li, Jian-Ying Ma, Wei Sun

**Affiliations:** ^1^Key Laboratory of Vegetation Ecology, Ministry of Education, Institute of Grassland Science, Northeast Normal University, Changchun, China; ^2^State Key Laboratory of Desert and Oasis Ecology, Xinjiang Institute of Ecology and Geography, Chinese Academy of Sciences, Urumqi, China

**Keywords:** dark respiration, photosynthetic ^13^C discrimination, post-photosynthetic isotope fractionation, C_3_ species, C_4_ species, water stress

## Abstract

Whether photosynthetic pathway differences exist in the amplitude of nighttime variations in the carbon isotope composition of leaf dark-respired CO_2_ (δ^13^C_l_) and respiratory apparent isotope fractionation relative to biomass (Δ_R,biomass_) in response to drought stress is unclear. These differences, if present, would be important for the partitioning of C_3_-C_4_ mixed ecosystem C fluxes. We measured δ^13^C_l_, the δ^13^C of biomass and of potential respiratory substrates and leaf gas exchange in one C_3_ (*Leymus chinensis*) and two C_4_ (*Chloris virgata* and *Hemarthria altissima*) grasses during a manipulated drought period. For all studied grasses, δ^13^C_l_ decreased from 21:00 to 03:00 h. The magnitude of the nighttime shift in δ^13^C_l_ decreased with increasing drought stress. The δ^13^C_l_ values were correlated with the δ^13^C of respiratory substrates, whereas the magnitude of the nighttime shift in δ^13^C_l_ strongly depended on the daytime carbon assimilation rate and the range of nighttime variations in the respiratory substrate content. The Δ_R,biomass_ in the C_3_ and C_4_ grasses varied in opposite directions with the intensification of the drought stress. The contribution of C_4_ plant-associated carbon flux is likely to be overestimated if carbon isotope signatures are used for the partitioning of ecosystem carbon exchange and the δ^13^C of biomass is used as a substitute for leaf dark-respired CO_2_. The detected drought sensitivities in δ^13^C_l_ and differences in respiratory apparent isotope fractionation between C_3_ and C_4_ grasses have marked implications for isotope partitioning studies at the ecosystem level.

## Introduction

Carbon isotope discrimination occurs during plant photosynthetic CO_2_ fixation, resulting in all higher plants being depleted in ^13^C in organic carbon relative to atmospheric CO_2_ (Farquhar and Sharkey, 1982). Because of the differences in anatomical structure and photosynthetic physiological processes, C_4_ photosynthesis discriminates less against ^13^CO_2_ than does C_3_ photosynthesis, which results in the ^13^C content in C_4_ plants (−6‰ to −19‰) being enriched compared to C_3_ species (−24‰ to −34‰) (Smith and Epstein, 1971; Farquhar et al., 1989). This large photosynthetic pathway difference in carbon isotope composition (δ^13^C) is useful for partitioning net ecosystem CO_2_ exchange into its components at both ecosystem and regional scales (Still et al., 2003; Zhang et al., 2006; Shimoda et al., 2009). In these applications, foliar δ^13^C is often used as a substitute for the δ^13^C of leaf dark-respired CO_2_. However, recent studies have shown that leaf dark-respired CO_2_ is often enriched in ^13^C compared to bulk biomass in both C_3_ and C_4_ plants (Bowling et al., 2008; Sun et al., 2010; Ghashghaie and Badeck, 2013). Because of high variability in the δ^13^C of leaf dark-respired CO_2_ (δ^13^C_l_), the magnitude of ^13^C enrichment (respired CO_2_ vs. bulk biomass) varies substantially at the diel timescale and is highly sensitive to changes in environmental conditions (Ghashghaie et al., 2003; Sun et al., 2010; Werner and Gessler, 2011). This fact highlights the importance of understanding the mechanisms of short-term variations in δ^13^C_l_ and incorporating this phenomenon into ecosystem carbon exchange partitioning.

Large diel variations in δ^13^C_l_ (up to 14.8‰) have been observed in various plant functional types, including grasses, herbs and trees (Hymus et al., 2005; Prater et al., 2006; Werner et al., 2009). Several mechanisms have been proposed to explain short-term shifts in δ^13^C_l_. First, daytime carbon assimilation is associated with a large variation in photosynthetic ^13^C discrimination, which may alter the carbon isotope composition of carbohydrates and subsequently influence δ^13^C_l_ (Ghashghaie et al., 2001; Sun et al., 2009). Second, shifts in the utilization of isotopically different respiratory substrates may alter the carbon isotope signature of leaf dark-respired CO_2_. For instance, respiratory substrate changes from ^13^C-enriched soluble carbohydrates to ^13^C-depleted lipids may cause ^13^C depletion in leaf dark-respired CO_2_ (Tcherkez et al., 2003). Third, the heterogeneous ^13^C distribution in hexose molecules (atoms 3 and 4 are more enriched in ^13^C than carbon atoms 1, 2, 5 and 6; Rossmann et al., 1991; Gleixner and Schmidt, 1997) and incomplete oxidation of glucose can cause up to a 4‰ shift in δ^13^C_l_ (Hobbie and Werner, 2004; Werner and Gessler, 2011). Glucose is converted to two molecules of pyruvate during glycolysis. Either the pyruvate can be completely oxidized to CO_2_ during the tricarboxylic acid (TCA) cycle or only two CO_2_ molecules are produced per glucose molecule and the molecules can be used for biosynthesis (e.g., acetyl-CoA). The CO_2_ produced in the latter case is enriched in δ^13^C (Rossmann et al., 1991; Gleixner and Schmidt, 1997). The magnitude of intra-molecular ^13^C differences in C_3_ species (on average, C-3 and C-4 are enriched in ^13^C by 6.2‰ compared to C-1, C-2, C-5 and C-6) is greater than that in C_4_ species (on average, C-3 and C-4 are enriched in ^13^C by 3.3‰ compared to C-1, C-2, C-5 and C-6), which may cause photosynthetic pathway differences in the magnitude of short-term variations in δ^13^C_l_. Finally, changes in the carbon isotope signature in malate and the contribution of malate decarboxylation (release of ^13^C-enriched CO_2_) to total respiratory CO_2_ flux may also result in short-term variations in δ^13^C_l_ (Barbour et al., 2007a, 2011; Gessler et al., 2009). In a recent study, Lehmann et al. (2015) reported strong correlations between δ^13^C_l_ and the δ^13^C of malate during both daytime and nighttime in potato plants growing under various temperature and soil moisture conditions. The aforementioned mechanisms are responsible for not only short-term variations in δ^13^C_l_ but also changes in respiratory apparent isotope fractionation (Ghashghaie et al., 2001, 2003; Bowling et al., 2008).

The carbon isotope signature and the magnitude of the diel shift in δ^13^C_l_ (maximum δ^13^C_l_ value – minimum δ^13^C_l_ value) differ substantially among plant functional types and are sensitive to changes in environmental conditions, such as, drought (Duranceau et al., 1999; Ghashghaie et al., 2001; Priault et al., 2009; Sun et al., 2010). In a study of *Phaseolus vulgaris*, Duranceau et al. (1999) reported that progressive drought alters not only the δ^13^C_l_ value but also nighttime variations in δ^13^C_l_. Compared to deep-rooted woody plants, δ^13^C_l_ in shallow-rooted grasses and herbs is more sensitive to seasonal variations in soil water availability in shallow soil layers (Sun et al., 2010). Plant functional type differences in the sensitivity of leaf gas exchange and plant growth to drought stress has been extensively reported, with deep-rooted trees and shrubs being less sensitive to drought than shallow-rooted herbs and grasses (Bucci et al., 2009; Comas et al., 2013). Recent studies have shown that the leaf net carbon assimilation rate in C_4_ grasses is equally or even more sensitive to drought stress compared to that in C_3_ grasses (Ripley et al., 2007, 2010). However, differences in drought sensitivity in the isotopic signatures of leaf dark-respired CO_2_ between C_3_ and C_4_ species remain largely unknown. This information is critical for the separation of C exchange in C_3_-C_4_ mixed ecosystems and assessing the drought sensitivity of component fluxes.

Using a pot experiment, we measured δ^13^C_l_, the δ^13^C values of bulk leaf tissue and potential respiratory substrates (soluble sugars, starch and lipids), the pool size of labile C substrates and leaf gas exchange in one C_3_ (*Leymus chinensis*) and two C_4_ (*Chloris virgata* and *Hemarthria altissima*) grasses during a manipulated drought period. We focused on the impacts of drought on the trend and range of nighttime shifts in δ^13^C_l_ in these dominant species of the meadow steppe in Northeast China. The carbon isotope composition of leaf dark-respired CO_2_ and the magnitude of nighttime variation in δ^13^C_l_ are predicted to be sensitive to manipulated drought treatments in both C_3_ and C_4_ grasses. Compared to the C_4_ grasses, the studied C_3_ grass is likely to have greater nighttime variations in δ^13^C_l_ because the magnitude of the intra-molecular ^13^C differences in C_3_ species is greater than that in C_4_ species.

## Materials and methods

### Experimental design and treatments

The experiment was performed at the Grassland Ecological Research Station of Northeast Normal University, Jilin Province, China (44°40′-44°44′N, 123°44′-123°47′E). The research station has a semi-arid, continental climate with mean annual temperature ranged from 4.6 to 6.4°C (1950–2004). Mean annual precipitation ranged from 280 to 644 mm (1950–2014) with over 70% of the precipitation occurs from June to August. Potential evapotranspiration is approximately three times that of the annual precipitation. Vegetation is dominated by *L. chinensis*, a C_3_ perennial rhizomatous grass; *Phragmites australis, C. virgata* and *H. altissima* are also abundant. Soil is classified as a chernozem soil, with 2.0% soil organic carbon content and 0.15% soil total nitrogen content (Wang et al., 2015).

One C_3_ perennial grass (*L. chinensis*) and two C_4_ grasses (annual: *C. virgata*; perennial: *H. altissima*) that co-occur in the meadow steppe of the study area were selected as experimental plants. *L. chinensis* is a widespread dominant grass of arid and semi-arid steppe in northern China, eastern Mongolia and Transbaikalia, Russia (Wang and Ba, 2008) and has an ability to resist drought, cold and alkaline conditions (Shi and Wang, 2005). *C. virgata* is widely distributed on the Northeast China Plain and is ecologically and economically important because of its high protein content and seed production. In addition, it also grows rapidly and is highly tolerant of alkaline conditions (Yang et al., 2008; Lin et al., 2016). *H. altissima* is a perennial rhizomatous grass and is distributed in tropical, subtropical and temperate regions, especially in China and Southeast Asia. It has strong adaptability and stress resistance and can be used as a good soil and water conservation crop (Han et al., 2016).

On DOY 135 in 2013, seedlings of *L. chinensis* and *H. altissima* were transplanted to plastic pots (23.5 cm in diameter and 20 cm in height) filled with chernozem soil (8 kg soil pot^−1^). For *C. virgata*, plants were germinated from seeds and transplanted to plastic pots. All species were planted as monocultures (five individuals per pot). Before the initiation of the drought treatment, all the transplanted plants were manually watered (to field capacity) every 3 days. To ensure that plant growth was not limited by nutrient elements, each pot received 2 mg of nitrogen fertilizer in the form of NH_4_NO_3_ every week. All the pots were watered thoroughly on the date (DOY 165) prior to the initiation of the drought treatment. During the drought experiment period (DOY 166–172), we stopped watering the plants. Moreover, all the pots were placed under a plastic shed to exclude natural precipitation. Variations in the soil water content for the studied grasses are provided in the supplementary information (Figure S1). The measurements of leaf gas exchange and collection of both leaf dark-respired CO_2_ and fresh materials were conducted on DOY 166 (day 1 of the experiment), DOY 168 (day 3 of the experiment), DOY 170 (day 5 of the experiment) and DOY 172 (day 7 of the experiment). Before the initiation of the drought treatment, the studied grasses were in the stem elongation stage with average heights of 51.6, 62.9 and 42.4 cm for *C. virgata, H. altissima* and *L. chinensis*, respectively. The tiller densities of the studied grasses were 36 pot^−1^, 19 pot^−1^ and 17 pot^−1^ for *C. virgata, H. altissima* and *L. chinensis*, respectively. Each pot had total leaf areas of 1,045 cm^2^ pot^−1^, 618 cm^2^ pot^−1^ and 648 cm^2^ pot^−1^ for *C. virgata, H. altissima* and *L. chinensis*, respectively.

### Leaf gas exchange measurements

Leaf gas exchange parameters (net CO_2_ assimilation rate, respiration rate, stomatal conductance, leaf-to-air vapor pressure deficit, leaf intercellular air space and ambient CO_2_ concentration) were measured every 3 h during a 24-h experimental cycle using an LI-6400 portable photosynthesis system (Li-Cor Biosciences, Lincoln, NE, USA). For each species, five pots were used for leaf gas exchange measurements. For each pot, two of the upper-most fully expanded leaves (the 2^nd^ or 3^rd^ leaf from the top) were measured for gas exchange parameters. The same leaves were marked and measured repeatedly throughout the experimental period. Before each measurement, the environmental conditions inside the leaf chamber (i.e., photosynthetically active radiation, air temperature, relative humidity and CO_2_ concentration) were set to match ambient conditions. The leaf respiration rate (*R*) was measured at 21:00, 00:00 and 03:00 h while the light intensity was set to zero.

### Meteorological data and soil water content

Air temperature, photosynthetic photon flux density (PPFD), relative humidity and air saturation vapor pressure were obtained from an eddy tower approximately 2 km away from the experimental site on days 1, 3, 5 and 7 of the drought treatment. Volumetric soil water contents (SWC-V) were measured using an ECH2O soil moisture sensor (EC-5, Decagon Ltd., Pullman, WA, USA) and the data were collected with a ProCheck device (Decagon Ltd., Pullman, WA, USA).

### Collection of leaf dark-respired CO_2_

Leaf dark-respired CO_2_ was collected using a 60-ml gas-tight syringe (Werner et al., 2007). Young and fully expanded leaves (comparable to those used for the leaf gas exchange measurements) were used for the collection of leaf dark-respired CO_2_. After the leaves (10–20 leaves) were placed inside the syringe, the syringe barrel was immediately flushed with CO_2_-free air five times by actuating the syringe plunger, and then, the leaf dark-respired CO_2_ was allowed to accumulate for 15 min in the syringe barrel. After the buildup of leaf-respired CO_2_, a 5-ml air sample containing leaf dark-respired CO_2_ was injected into a helium-flushed 12-ml vial (Presentation 1). Leaf dark-respired CO_2_ was collected during the nighttime period (21:00, 00:00 and 03:00 h) for each of the four sampling dates. For each sampling time, leaf dark-respired CO_2_ collection was repeated on five pots for each of the three studied grasses.

### Extraction of lipids, soluble sugars and starch

For each sampling date, leaves comparable to those used for the leaf-respired CO_2_ sampling and gas exchange measurements were collected at 21:00, 00:00 and 03:00 h. The collected leaves were immediately flash frozen in liquid nitrogen to stop respiratory metabolic activities, temporarily stored in a deep freezer and then freeze-dried in a Labconco freeze drier (Labconco, Kansas City, MO, USA). The freeze-dried leaves were ground to a fine powder using a ball mill (MM 400 Retsch, Haan, Germany). Lipids, soluble sugars and starch were extracted using the protocols described by Wanek et al. (2001) and Göttlicher et al. (2006). In brief, the powdered leaf material (100 mg) was extracted with 1 ml of methanol/chloroform/water (MCW; 12:5:3, v/v/v) for 30 min at 70°C. After cooling, the samples were centrifuged at 10,000 g for 2 min. The supernatant (0.65 ml) was transferred to a new vial and phases were separated by adding 0.2 ml of chloroform and 0.7 ml of water. To determine the carbon isotope ratio of the lipids, 50 μl of the chloroform phase as pipetted into smooth tin capsules for liquids (4.75 × 11 mm, Santis Analytical AG, Teufen, Switzerland) and dried under a fume hood. Samples were then analyzed by an isotope ratio mass spectrometer (described below). Chlorophyll was also extracted with lipids, which may cause overestimation of the ^13^C content of lipids (Bidigare et al., 1991). The methanol/water phase (upper layer) was removed and processed for sugar isolation. Soluble sugars were isolated using an ion exchange column containing cation-exchange resin (Dowex 50 W × 8, Sigma Aldrich, St. Louis, MO, USA) and anion-exchange resin (Dowex 1 × 8, Sigma Aldrich, St. Louis, MO, USA). The columns were rinsed with deionized water, and effluent was dried in tin capsules for carbon isotope ratio measurement of soluble sugars. The pellet from the centrifugation was rinsed with deionized water, oven-dried after re-extraction with MCW three times, and then heated to 100°C to gelatinize the starch. After cooling to room temperature, a heat-stable α-amylase solution (A3306, Sigma-Aldrich, St. Louis, MO, USA) was added, and the samples were incubated at 85°C for 120 min. Thereafter, the samples were cooled to room temperature and centrifuged at 10,000 g for 3 min. The supernatant was transferred to pre-washed centrifugal ultrafiltration devices (Microcon YM-10, Millipore, Billerica, MA, USA) to remove the enzymes and other high molecular weight substances. The filtrates were then dried in a tin capsule to analyze the carbon isotope composition of the starch.

### Isotope ratio mass analysis

All carbon isotope ratio analyses were performed using an isotope ratio mass spectrometer (Isoprime 100, Elementar, Langenselbold, Germany) coupled to an elemental analyzer (vario EL cube, Elementar, Langenselbold, Germany) for solid samples or a Trace Gas Pre-concentrator (Elementar, Langenselbold, Germany) for gaseous samples. The precision of repeated δ^13^C measurements on the working standards of solid and gaseous substrates was <0.1‰. The carbon isotope composition of the working standards was calibrated using reference materials from IAEA. The identical treatment principle was applied during the preparation of samples, checking standards and working standards. The natural abundance of ^13^C in the samples is reported relative to VPDB as follows:

(1) $$\begin{array}{l} \delta^{13}\text{C}\left(‰\right)\;=\;{\text{R}}_{\text{sample}}/R_{\text{standard}}-1 \end{array}$$

### δ^13^C of the leaf photosynthate pool

The δ^13^C of the cumulative photosynthate pool was estimated as follows:

(2) $$\begin{array}{l} \delta^{13}C_{pw}=\frac{\overset{n}{\underset{i=1}{\sum }}A_{i}\times \delta^{13}C_{pi}}{\overset{n}{\underset{i=1}{\sum }}A_{i}} \end{array}$$

where δ^13^C_pw_ is the assimilation-weighted, cumulative carbon isotopic value of the recently fixed photosynthates and *A*_i_ and δ^13^C_*pi*_ are the instantaneous net assimilation rate and the δ^13^C value of photosynthates at time *i* (06:00, 09:00, 12:00, 15:00 and 18:00 h), respectively. Carbon isotope composition of photosynthates (δ^13^C_*p*_) was solved using the following equation:

(3) $$\begin{array}{l} \Delta_{p}=\frac{\delta^{13}C_{a}-\delta^{13}C_{p}}{1+\delta^{13}C_{p}} \end{array}$$

where δ^13^C_a_ represents δ^13^C signature of atmospheric CO_2_ (assumed to be −8‰) and Δ_*P*_ represents photosynthetic discrimination against ^13^C.

For C_3_ species, Δ_*P*_ was estimated from the simplified linear model of Farquhar et al. (1982) as follows:

(4) $$\begin{array}{l} \Delta_{p}=a+\left(b-a\right)C_{i}/C_{a} \end{array}$$

where *a* represents isotope fractionation occurring during diffusion through the air (4.4‰); *b* represents the net isotope fractionation associated with carboxylation reaction of ribulose-1,5-bisphosphate carboxylase/oxygenase (Rubisco) (27‰); and *C*_*i*_ and *C*_*a*_ are the leaf intercellular air space and ambient CO_2_ concentrations, respectively. For the parameters in the Equation (4): *a* and *b* were obtained from literature (Farquhar et al., 1982) and *C*_*i*_ and *C*_*a*_ were measured using the LI-6400 portable photosynthesis system.

For the C_4_ species, Δ_*p*_ was estimated from a modified equation of Farquhar (1983) model of ^13^C discrimination during C_4_ plant photosynthesis as follows (Farquhar, 1983; Peisker and Henderson, 1992):

(5) $$\begin{array}{l} \Delta_{p}=a+\left(b_{4}+\phi \left(b_{3}-s\right)-a\right)C_{i}/C_{a} \end{array}$$

where *a* represents isotope fractionation associated with the diffusion of CO_2_ into the leaf (4.4‰); *b*_4_ represents net isotope fractionation associated with the dissolution of CO_2_ to ${\text{HCO}}_{3}^{-}$ (−7.9‰) and fixation by phosphoenolpyruvate carboxylase (PEPc, 2.2‰); ^13^C fractionation associated with the dissolution of CO_2_ to ${\text{HCO}}_{3}^{-}$ is temperature dependent; therefore, *b*_4_ was calculated for each time point using a temperature-correction equation provided by Mook et al. (1974); *b*_3_ represents isotope fractionation associated with Rubisco carboxylation (29‰); *s* represents isotope fractionation associated with the diffusion of CO_2_ out of the bundle sheath cells (1.8‰); φ represents the leakiness of the bundle sheath to CO_2_; and *C*_*i*_ and *C*_*a*_ represent the leaf intercellular air space and ambient CO_2_ concentrations, respectively. It has been reported that φ changes with plant water status (Buchmann et al., 1996; Williams et al., 2001; Tazoe et al., 2008; Sun et al., 2012); therefore, we assumed the leakiness on day 1 (optimum soil water condition) is 0.2 and on day 7 (severe drought stress) is 0.4 and generated a linear relationship between stomatal conductance and leakiness, which was used to estimate the leakiness on day 3 and 5. For the parameters in the Equation (5): *a* and *b*_3_ were obtained from literature (Farquhar, 1983), *b*_4_ and φ were calculated according to the environmental conditions and *C*_*i*_ and *C*_*a*_ were measured using the LI-6400 portable photosynthesis system.

### Respiratory apparent ^13^C/^12^C fractionation

Respiratory apparent isotope fractionation relative to a particular substrate X (e.g., leaf bulk materials, starch, soluble sugars, lipids and cumulative photosynthate pool) was calculated as follows:

(6) $$\begin{array}{l} \Delta_{R,X}=\frac{\delta^{13}C_{X}-\delta^{13}C_{l}}{1+\delta^{13}C_{l}} \end{array}$$

where δ^13^C_X_ is the δ^13^C signature of substrate X and δ^13^C_l_ is δ^13^C of the leaf dark-respired CO_2_.

### Measurements of the concentration of soluble sugars and transitory starch

Concentrations of soluble sugars and transitory starch were measured according to the microplate enzymatic method (Zhao et al., 2010). Briefly, soluble sugars were extracted by adding ethanol (EtOH) to powered plant tissue and heating in a water bath at 80°C. Each sample was extracted three times, and supernatants were purified by adding activated charcoal (Sigma C7606). After centrifugation, aliquots (20 μl) of soluble sugar extract were transferred to a 96-well microplate and placed in an oven to remove EtOH. Glucose, fructose and sucrose were assayed sequentially using the same sample. After the addition of 20 μl of DI water and a 100 μl mixture of the glucose assay reagent (Hexokinase, Sigma G3293), the microplates were incubated at 30°C for 15 min and measured for absorbance at 340 nm using a SpectraMax Plus Microplate Reader (Molecular Devices Corporation, Sunnyvale, CA, USA). The glucose concentration was calculated using a glucose standard curve. The fructose and sucrose concentrations were measured by changes in glucose concentration after adding 10 μl of phosphoglucose isomerase (Sigma P9544**-**0.25 EU) and 10 μl of invertase (Sigma I4504-83 EU) solution, respectively.

Starch was extracted by adding KOH to the sample residue remaining after the EtOH extraction of leaf soluble sugars and heating in a boiling water bath for 1 h. After the neutralization of KOH, Tris buffer and α-amylase solution (Sigma A3403) were added, and the test tube was incubated at 85°C for 30 min. To complete the starch hydrolysis, 1 ml of amyloglucosidase (Sigma A7095) was added, and the test tube was incubated at 55°C for 60 min and then placed in a boiling water bath for 4 min. Eventually, DI water was used to bring the test tube to a final volume of 6 ml, and the supernatant (centrifuged at 3,000 g for 10 min) was assayed for glucose as previously described. To account for water loss when glucose units are linked to form starch, the starch concentration in the sample was calculated according to the glucose concentration in the tissue and residue multiplied by a factor of 0.9.

### Statistical analysis

One-way analysis of variance (ANOVA) was used to assess drought effects on diurnal average leaf gas exchange parameters, the carbon isotope composition of leaf dark-respired CO_2_ (δ^13^C_l_), leaf soluble sugars (δ^13^C_soluble_ _sugar_), leaf starch (δ^13^C_starch_) and leaf lipids (δ^13^C_lipid_). Two-way ANOVA was used to assess nighttime variation and drought impacts on δ^13^C_l_, δ^13^C_soluble_ _sugar_, δ^13^C_starch_ and δ^13^C_lipid_, as well as on soluble sugars and starch concentrations. Linear regression analysis was used to assess correlations between δ^13^C_l_ and δ^13^C_soluble_ _sugar_, δ^13^C_starch_, δ^13^C_lipid_ and δ^13^C_pw_. Linear regression analysis was also conducted to evaluate dependence of the amplitude of nocturnal shifts in δ^13^C_l_ on the diurnal average net CO_2_ assimilation rate, nighttime average respiration rate, diurnal average stomatal conductance, diurnal average of the C_i_/C_a_ ratio and diurnal average vapor pressure deficit, as well as on the magnitude of nighttime variations in leaf soluble sugars and starch concentrations. All analyses were carried out using SPSS software version 22 (SPSS Inc., Chicago, IL, USA). Average values are reported as arithmetic mean ± 1 SE.

## Results

### Leaf gas exchange

**Figure 1:**
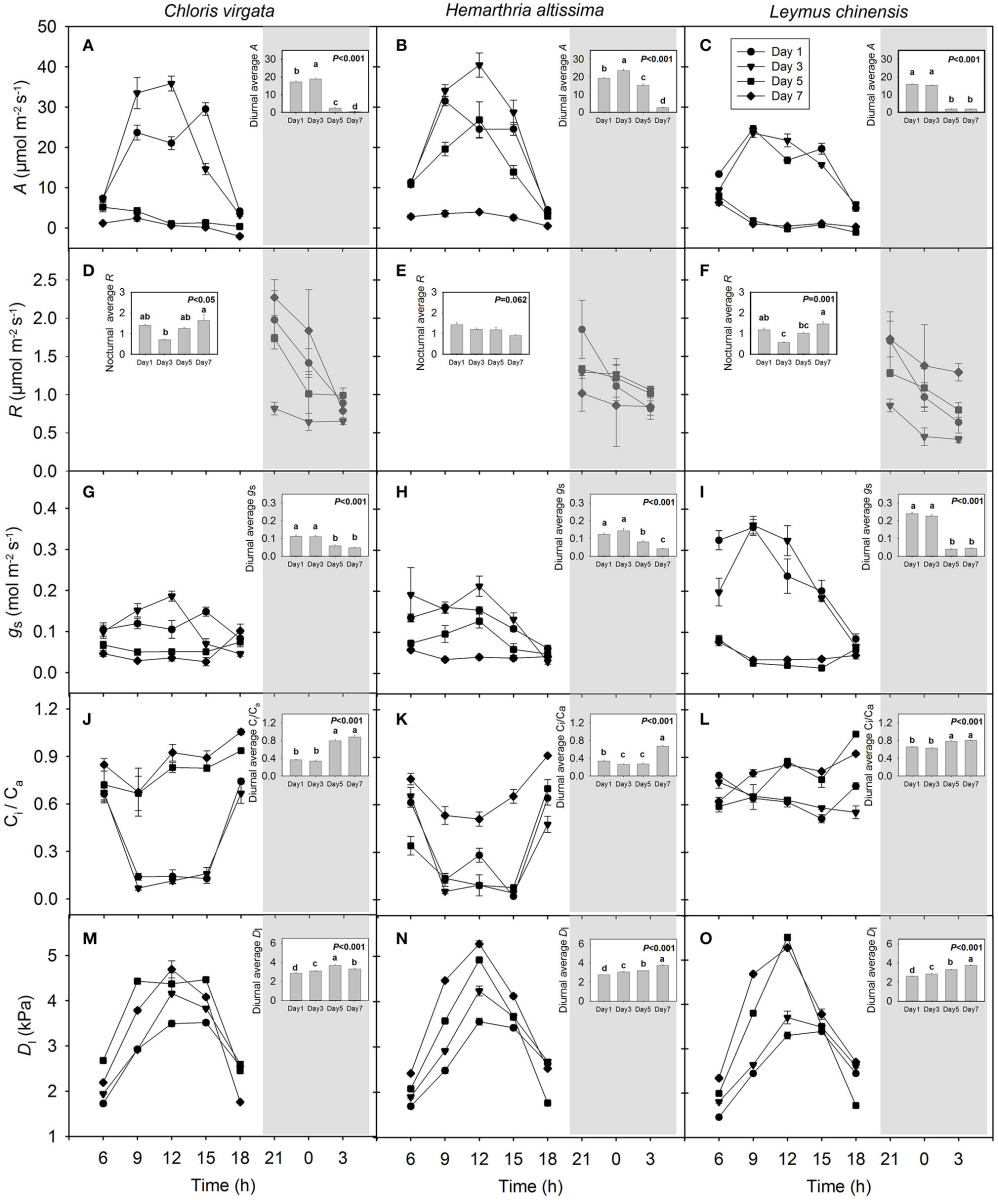
Variations of **(A–C)** leaf net CO_2_ assimilation rate (*A*; μmol m^−2^ s^−1^), **(D–F)** respiration rate (*R*; μmol m^−2^ s^−1^), **(G–I)** stomatal conductance (*g*_*s*_; mol m^−2^ s^−1^), **(J–L)** C_i_/C_a_ ratio (unitless) and **(M–O)** leaf-to-air vapor pressure deficit (*D*_l_; kPa) on the day 1 (circle), day 3 (triangle), day 5 (square) and day 7 (diamond) of the drought treatment in *Chloris virgata* (annual C_4_), *Hemarthria altissima* (perennial C_4_) and *Leymus chinensis* (perennial C_3_). Diurnal average values are presented as an inset figure. Different lowercase letters in the inset figures indicate significant differences (*P* < 0.05) between the sampling dates (Tukey's test). Data are reported as the arithmetic mean ± 1 standard error (*n* = 5). The shaded area denotes the dark period.

Large diurnal variations in the net carbon assimilation rate (*A*) were detected on days 1 and 3, but not on days 5 and 7, especially in the C_4_ annual grass *C*. *virgata* and the C_3_ perennial grass *L. chinensis* (Figures 1A–C). Diurnal average *A* decreased by 97.2%, 86.0% and 88.4% in *C*. *virgata, H. altissima* and *L. chinensis*, respectively, with the progress of drought stress and differed significantly among the measurement dates (Figures 1A–C). For each measurement date, the leaf nighttime respiration rate (*R*) decreased from 21:00 to 03:00 h (Figures 1D–F). For *H. altissima*, the nighttime average *R* decreased by 36.5% with the intensification of drought stress, whereas the nighttime average *R* in *C*. *virgata* and *L. chinensis* increased by 131.8% and 155.4%, respectively, from day 3 to 7. Strong diurnal variations in stomatal conductance (*g*_s_) were observed in *L*. *chinensis* on days 1 and 3, but not on days 5 and 7 (Figure 1I). The C_i_/C_a_ ratio varied substantially on the diurnal timescale especially in *C. virgata* on days 1 and 3 and in *H. altissima* on days 1, 3 and 5 (Figures 1J–L). The diurnal average C_i_/C_a_ ratio increased from 0.36 ± 0.02, 0.34 ± 0.02 and 0.65 ± 0.01 on day 1 to 0.88 ± 0.04, 0.67 ± 0.01 and 0.80 ± 0.01 on day 7 for *C. virgata, H. altissima* and *L. chinensis*, respectively (Figures 1J–L). The leaf-to-air vapor pressure deficit (*D*_l_) varied substantially in all studied grasses, with maximum values detected between 09:00 and 12:00 h (Figures 1M–O). For all species, the diurnal average *D*_l_ (kPa) increased from 2.84 ± 0.03, 2.76 ± 0.01 and 2.61 ± 0.02 on day 1 to 3.30 ± 0.05, 3.76 ± 0.02 and 3.76 ± 0.01 on day 7 for *C. virgata, H. altissima* and *L. chinensis*, respectively (Figures 1M–O).

### Variation in the δ^13^C of leaf dark-respired CO_2_

**Figure 2:**
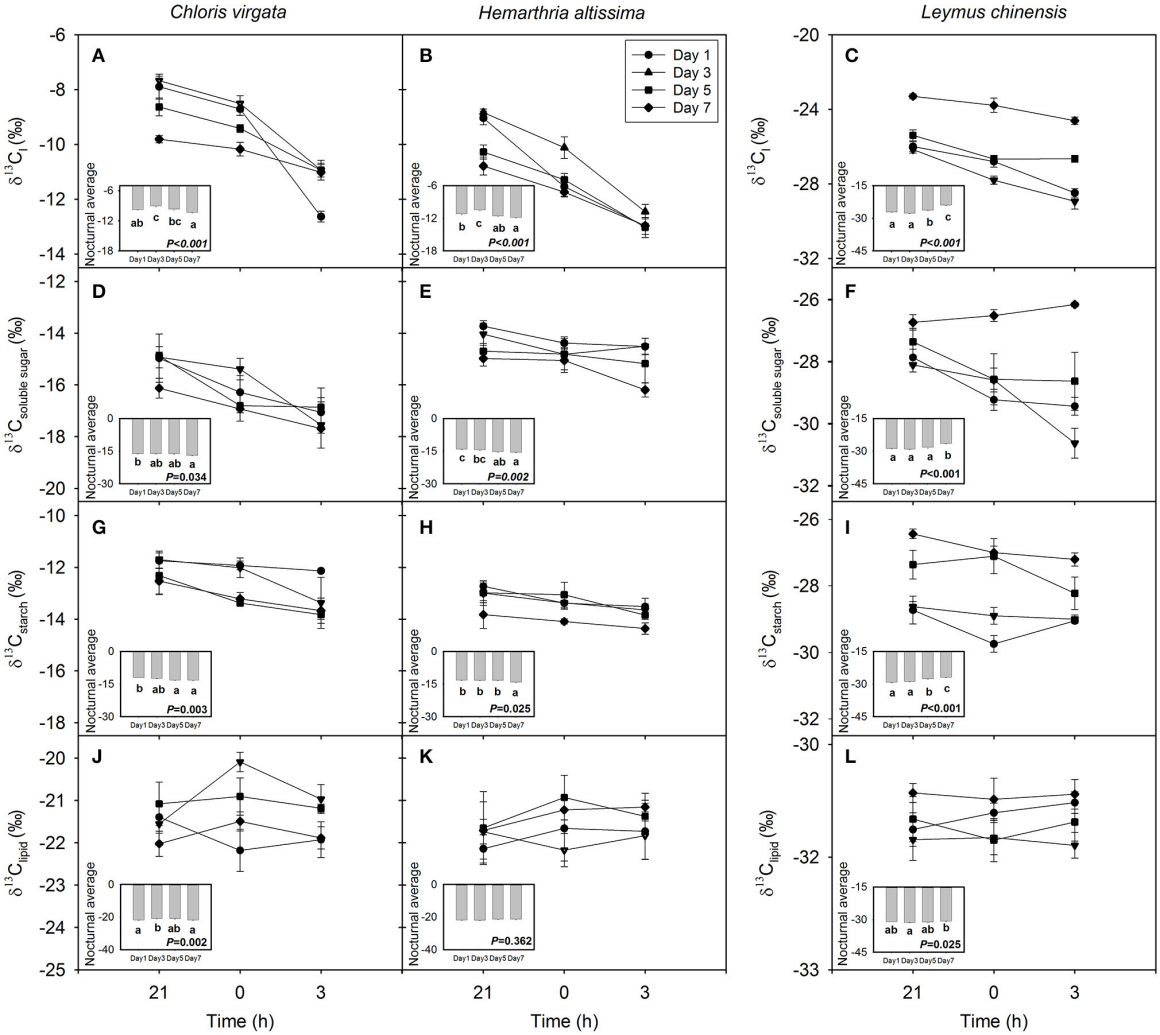
Nighttime variations in the C isotope composition (‰) of **(A–C)** leaf dark-respired CO_2_ (δ^13^C _l_), **(D–F)** leaf soluble sugars (δ^13^C_soluble_ _sugar_), **(G–I)** leaf starch (δ^13^C_starch_) and **(J–L)** leaf lipids (δ^13^C_lipid_) on the day 1 (circle), day 3 (triangle), day 5 (square) and day 7 (diamond) of the drought treatment in *Chloris virgata* (annual C_4_), *Hemarthria altissima* (perennial C_4_) and *Leymus chinensis* (perennial C_3_). Diurnal average values are presented as an inset figure. Different lowercase letters in the inset figures indicate significant differences (*P* < 0.05) between the sampling dates (Tukey's test). Data are reported as the arithmetic mean ± 1 standard error (*n* = 5).

For all studied grasses, the ^13^C content in leaf dark-respired CO_2_ was depleted from 21:00 to 03:00 h; however, the magnitude of the nocturnal shift in the carbon isotope composition of leaf dark-respired CO_2_ (δ^13^C_l_) decreased with the intensification of drought stress (Figures 2A–C). Nighttime average δ^13^C_l_ differed significantly among the sampling dates (Figures 2A–C; Table 1), with nighttime average δ^13^C_l_ values declining from −9.7 ± 0.2‰ and −11.2 ± 0.2‰ on day 1 to −10.3 ± 0.1‰ and −11.8 ± 0.1‰ on day 7 for *C. virgata* and *H. altissima*, respectively. For the C_3_ grass *L. chinensis*, nighttime average δ^13^C_l_ values increased from −27.1 ± 0.2‰ on day 1 to −23.9 ± 0.2‰ on day 7 (Figure 2C).

**Table 1:** The *df* and *P* values from the two-way analysis of nighttime variation (N) and drought effects (D) in the C isotope composition of leaf-respired CO_2_ (δ^13^C_l_), leaf soluble sugars (δ^13^C_soluble_ _sugar_), leaf starch (δ^13^C_starch_) and leaf lipids (δ^13^C_lipid_) in *Chloris virgata* (annual C_4_), *Hemarthria altissima* (perennial C_4_) and *Leymus chinensis* (perennial C_3_).

**Species**		**δ^13^C_l_ (‰)**		**δ^13^C_soluble__sugar_ (‰)**		**δ^13^C_starch_ (‰)**		**δ^13^C_lipid_ (‰)**	
		***df***	***P***	***df***	***P***	***df***	***P***	***df***	***P***
*Chloris virgata*	D	3	<0.001	3	0.158	3	0.001	3	0.002
	N	2	<0.001	2	<0.001	2	0.032	2	0.33
	D × N	6	<0.001	6	0.613	6	0.806	6	0.159
*Hemarthria altissima*	D	3	<0.001	3	0.003	3	0.005	3	0.391
	N	2	<0.001	2	0.037	2	0.012	2	0.66
	D × N	6	0.014	6	0.674	6	0.952	6	0.953
*Leymus chinensis*	D	3	<0.001	3	<0.001	3	<0.001	3	0.039
	N	2	<0.001	2	0.002	2	0.026	2	0.98
	D × N	6	0.008	6	0.023	6	0.144	6	0.817

### δ^13^C of potential respiratory substrates

The values of the carbon isotope composition of leaf soluble sugars (δ^13^C_soluble_ _sugar_) and starch (δ^13^C_starch_) decreased from 21:00 to 03:00 h except for δ^13^C_soluble_ _sugar_ in *L*. *chinensis* on day 7 (Figures 2D–I). There were strong drought impacts on nighttime average δ^13^C_soluble_ _sugar_ and nighttime average δ^13^C_starch_. Nighttime average δ^13^C_soluble_ _sugar_ and nighttime average δ^13^C_starch_ values decreased from −16.1 ± 0.2‰ and −11.9 ± 0.1‰ on day 1 to −16.9 ± 0.2‰ and −13.1 ± 0.3‰ on day 7 in *C. virgata* and from −14.1 ± 0.2‰ and −13.2 ± 0.2‰ on day 1 to −15.5 ± 0.2‰ and −14.1 ± 0.2‰ on day 7 in *H. altissima*. However, nighttime average δ^13^C_soluble_ _sugar_ and nighttime average δ^13^C_starch_ values increased from −28.8 ± 0.2‰ and −29.2 ± 0.1‰ on day 1 to −26.5 ± 0.1‰ and −26.9 ± 0.1‰ on day 7 in *L. chinensis* (Figures 2D–I; Table 1). No consistent nighttime variation patterns were observed in the δ^13^C of leaf lipids (δ^13^C_lipid_) (Figures 2J–L). Significant drought treatment impacts on the nighttime average δ^13^C_lipid_ were detected in *C*. *virgata* and *L*. *chinensis*, but not in *H*. *altissima* (Table 1).

### Respiratory apparent isotope fractionation

**Figure 3:**
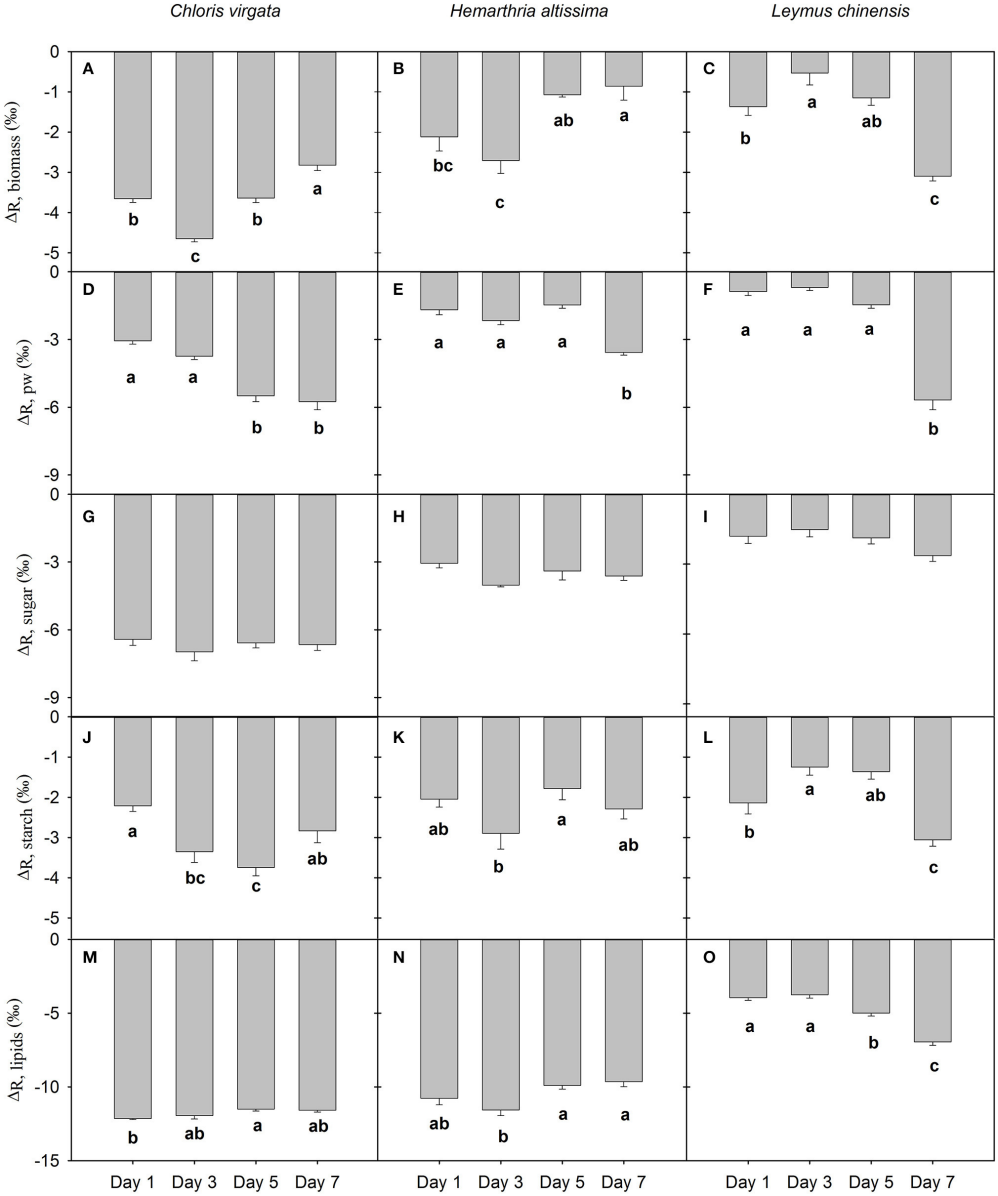
Respiratory apparent ^13^C/^12^C fractionation (‰) of leaf dark-respired CO_2_ relative to **(A–C)** biomass (Δ_R_, _biomass_), **(D–F)** recently fixed photosynthates (Δ_R_, _pw_), **(G–I)** soluble sugars (Δ_R_, _sugar_), **(J–L)** starch (Δ_R_, _starch_) and **(M–O)** lipids (Δ_R_, _lipids_) and on the days 1, 3, 5 and 7 of the drought treatment **(D)** in *Chloris virgata* (annual C_4_), *Hemarthria altissima* (perennial C_4_) and *Leymus chinensis* (perennial C_3_). The minus sign shows that the leaf dark-respired CO_2_ is ^13^C enriched compared to the potential respiratory substrates. Different lowercase letters indicate significant differences (*P* < 0.05) between the sampling dates (Tukey's test). Data are reported as the arithmetic mean ± 1 standard error (*n* = 5).

For the studied species, leaf dark-respired CO_2_ was enriched in ^13^C compared to biomass and other potential respiratory substrates (Figure 3). Respiratory apparent isotope fractionation relative to biomass (Δ_R,biomass_) differed significantly among the sampling dates for all studied species (Figures 3A–C). With the intensification of the water deficit, the magnitude of ^13^C enrichment in leaf dark-respired CO_2_ (relative to biomass) gradually decreased in the C_4_ grasses, whereas it increased in the C_3_ grass *L. chinensis* (Figure 3). Compared to the recently fixed photosynthates, leaf dark-respired CO_2_ was gradually enriched in ^13^C content in both C_3_ and C_4_ gasses as water deficit intensified (Figures 2D–F). Respiratory apparent isotope fractionation relative to photosynthates (Δ_R,pw_) changed from −3.1 ± 0.1‰, −1.7 ± 0.2‰ and −0.9 ± 0.2‰ on day 1 to −5.7 ± 0.4‰, −3.6 ± 0.1‰ and −5.7 ± 0.4‰ on day 7 for *C. virgata, H. altissima* and *L. chinensis*, respectively. There were no sampling date effects on respiratory apparent isotope fractionation relative to soluble sugars (Δ_R,sugar_); however, Δ_R,sugar_ values differed significantly between the C_3_ and C_4_ grasses (Figures 3G–I). We detected significant differences in respiratory apparent isotope fractionation relative to starch (Δ_R,starch_) between the sampling dates; however, there were no apparent photosynthetic pathway differences (Figures 3J–L). For all studied species, respiratory apparent isotope fractionation relative to lipids (Δ_R,lipid_) differed significantly between the sampling dates. Moreover, the magnitude of ^13^C enrichment in leaf dark-respired CO_2_ (relative to lipids) was greater in the studied C_4_ grasses (average Δ_R,lipid_ values of −11.8 ± 0.2‰ and −10.5 ± 0.5‰ for *C. virgata* and *H. altissima*, respectively) than in the C_3_ grass *L. chinensis* (an average Δ_R,lipid_ value of −4.9 ± 0.6‰; Figures 3M–O).

### Changes in the concentrations of soluble sugars and transitory starch

**Figure 4:**
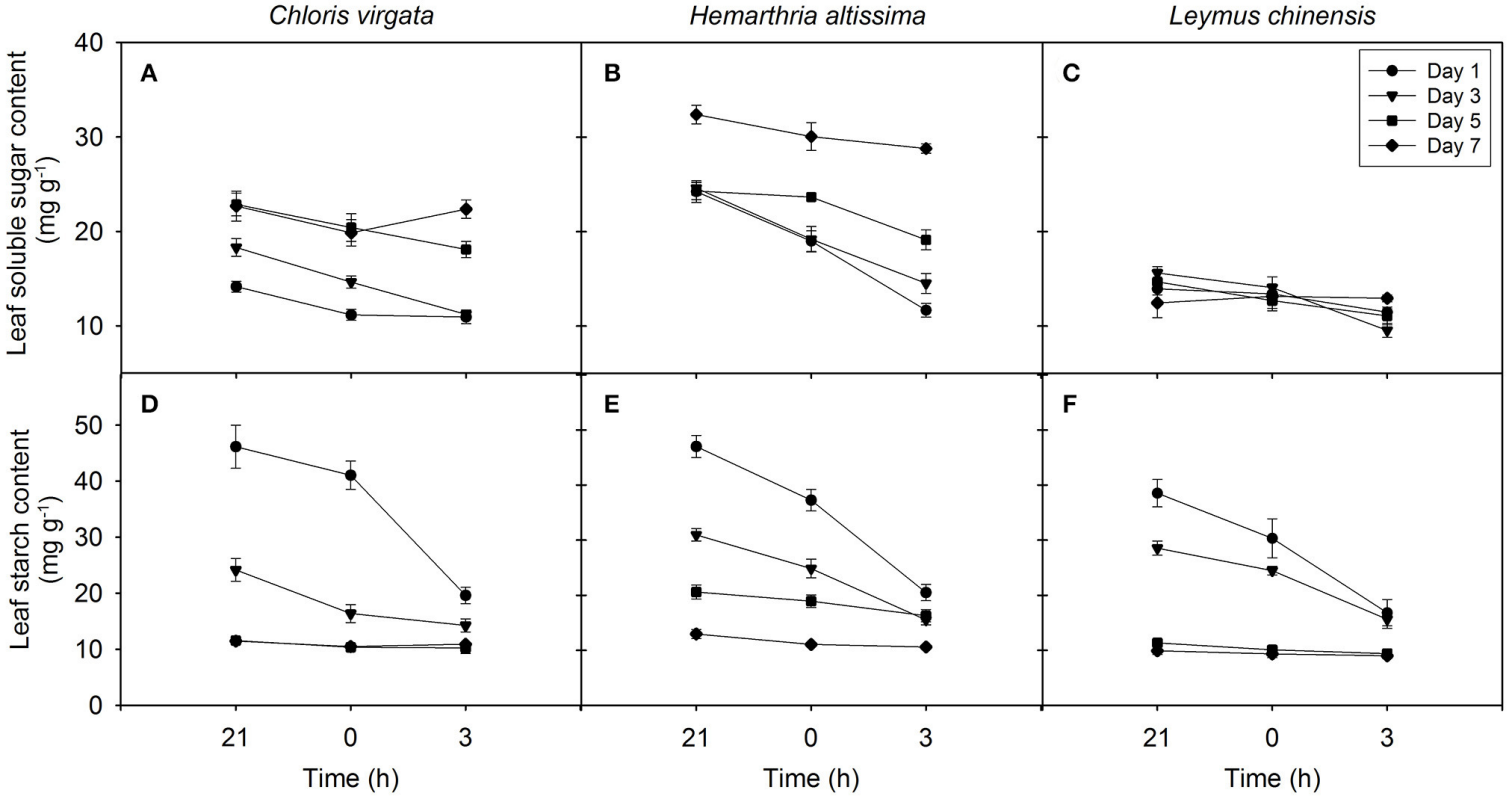
Nighttime variations in the content of leaf soluble sugars **(A–C)** and leaf starch **(D–F)** on the day 1 (circle), day 3 (triangle), day 5 (square), day 7 (diamond) of the drought treatment in *Chloris virgata* (annual C_4_), *Hemarthria altissima* (perennial C_4_), and *Leymus chinensis* (perennial C_3_). Data are reported as the arithmetic mean ± 1 standard error (*n* = 5).

Leaf soluble sugars showed a trend of decreasing from 21:00 to 03:00 h in all studied species; however, the range of nighttime variations in soluble sugars content (maximum value – minimum value) in the C_4_ grasses was greater than that in the C_3_ grass (Figures 4A–C). The impact of drought on the content of leaf soluble sugars was significant only in the C_4_ grasses (Table 2). For all studied species, the leaf starch content decreased from 21:00 to 0300 h, and the range of nighttime variations in starch content gradually diminished with the intensification of drought stress (Figures 4D–F). A significant drought effect on the leaf starch content was detected in all studied grasses (Table 2). Information on the content of the major sugars (glucose, fructose and sucrose) are provided in the supplementary section (Table S3).

**Table 2:** The *df* and *P* values from the two-way analysis of nighttime variation (N) and drought effects (D) on leaf soluble sugars content (mg g^−1^) and leaf starch content (mg g^−1^) in *Chloris virgata* (annual C_4_), *Hemarthria altissima* (perennial C_4_) and *Leymus chinensis* (perennial C_3_).

**Species**		**Leaf soluble sugar (mg g**^**−1**^**)**		**Leaf starch (mg g**^**−1**^**)**	
		***df***	***P***	***df***	***P***
*Chloris virgata*	D	3	<0.001	3	<0.001
	N	2	<0.001	2	<0.001
	D × N	6	0.032	6	<0.001
*Hemarthria altissima*	D	3	<0.001	3	<0.001
	N	2	<0.001	2	<0.001
	D × N	6	<0.001	6	<0.001
*Leymus chinensis*	D	3	0.985	3	<0.001
	N	2	<0.001	2	<0.001
	D × N	6	0.044	6	<0.001

### Correlation between δ^13^C_l_ and the δ^13^C of respiratory substrates

**Figure 5:**
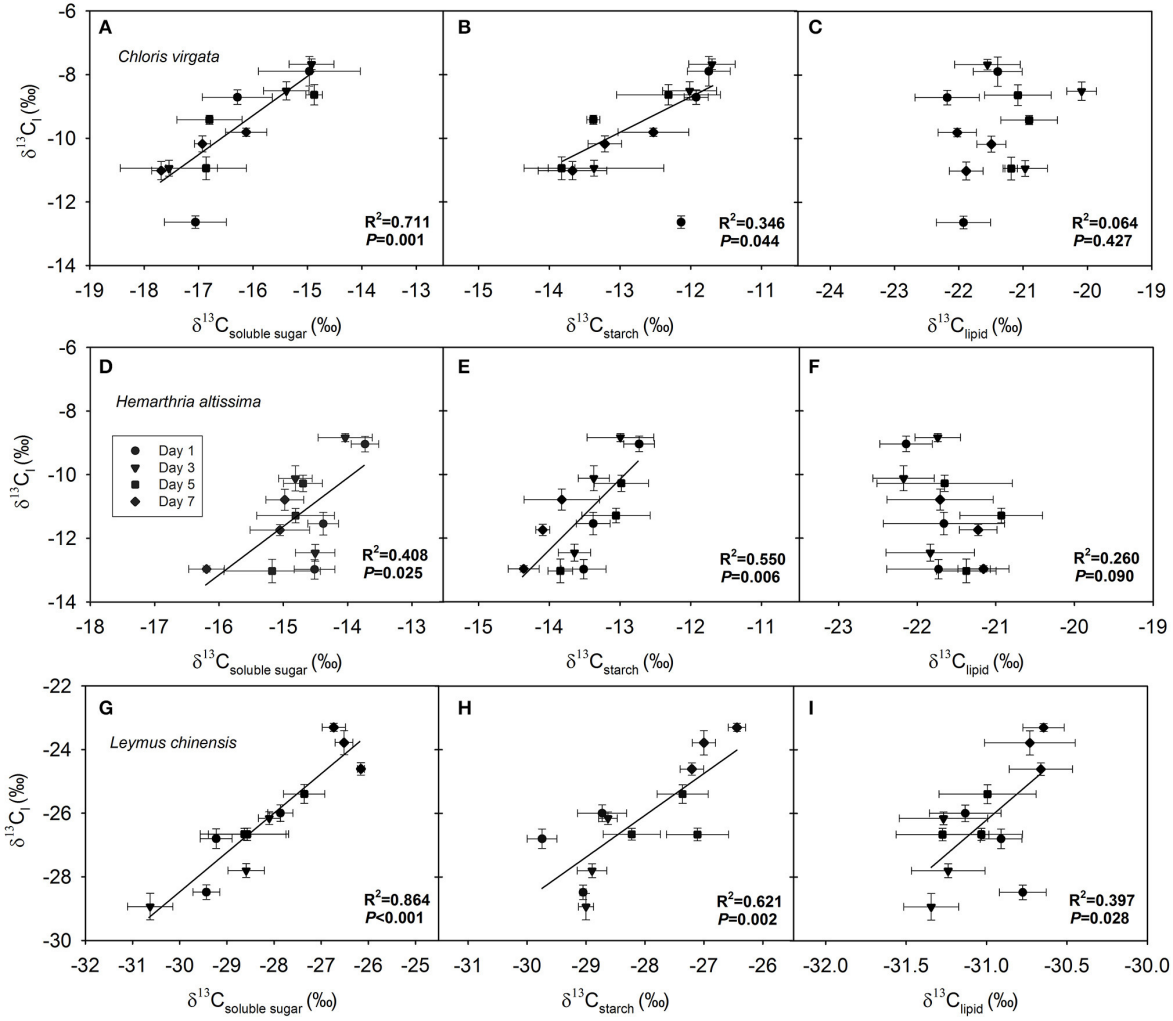
Dependence of the C isotope composition of leaf dark-respired CO_2_ (δ^13^C_l_) on **(A,D,G)** δ^13^C of leaf soluble sugars (δ^13^C_soluble_ _sugar_), **(B,E,H)** δ^13^C of leaf starch (δ^13^C_starch_) and **(C,F,I)** δ^13^C of leaf lipids (δ^13^C_lipid_) in *Chloris virgata* (annual C_4_), *Hemarthria altissima* (perennial C_4_) and *Leymus chinensis* (perennial C_3_). The *P* values and *R*^2^ of the linear relationship are provided.

After pooling data across the four measurement dates, we detected a strong dependence of δ^13^C_l_ on δ^13^C_soluble_ _sugar_ (Figures 5A,D,G) and δ^13^C_starch_ (Figures 5B,E,H) in all studied grasses. However, a significant correlation between δ^13^C_l_ and δ^13^C_lipid_ was detected only in the C_3_ species *L*. *chinensis* (Figure 5I), and not in the C_4_ species *C*. *virgata* and *H*. *altissima* (Figures 5C,F).

### Dependence of the magnitude of the nocturnal shift in δ^13^C on gas exchange parameters and variations in substrate availability

**Figure 6:**
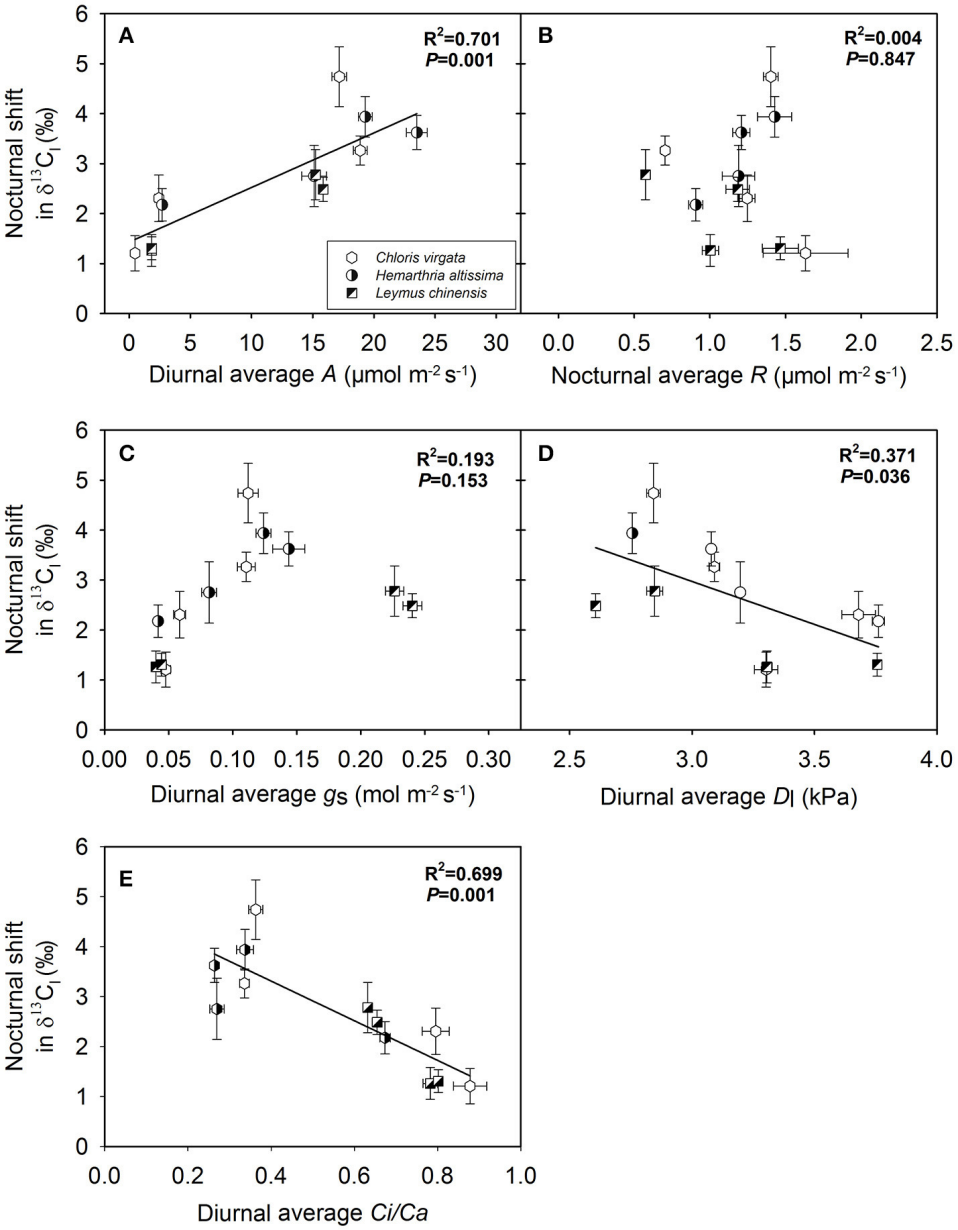
Dependence of the magnitude of the nocturnal shift in the C isotope composition of leaf dark-respired CO_2_ (δ^13^C_l_) on **(A)** diurnal average leaf net CO_2_ assimilation rate (diurnal average *A*; μmol m^−2^ s^−1^), **(B)** nighttime average leaf respiration rate (nocturnal average R; μmol m^−2^ s^−1^), **(C)** diurnal average stomatal conductance (diurnal average *g*_*s*_; mol m^−2^ s^−1^), **(D)** diurnal average leaf-to-air vapor pressure deficit (diurnal average *D*_l_; kPa) and **(E)** diurnal average of the C_i_/C_a_ ratio (diurnal average of the C_i_/C_a_; unitless) in *Chloris virgata* (hexagon, annual C_4_), *Hemarthria altissima* (semi-filled circle, perennial C_4_), and *Leymus chinensis* (diagonal-filled square, perennial C_3_). The amplitude of nocturnal shift in δ^13^C_l_ was calculated as δ^13^C_l_ values at 21:00 h minus δ^13^C_l_ values at 03:00 h. The *P* values and *R*^2^ of the linear relationship are provided.

**Figure 7:**
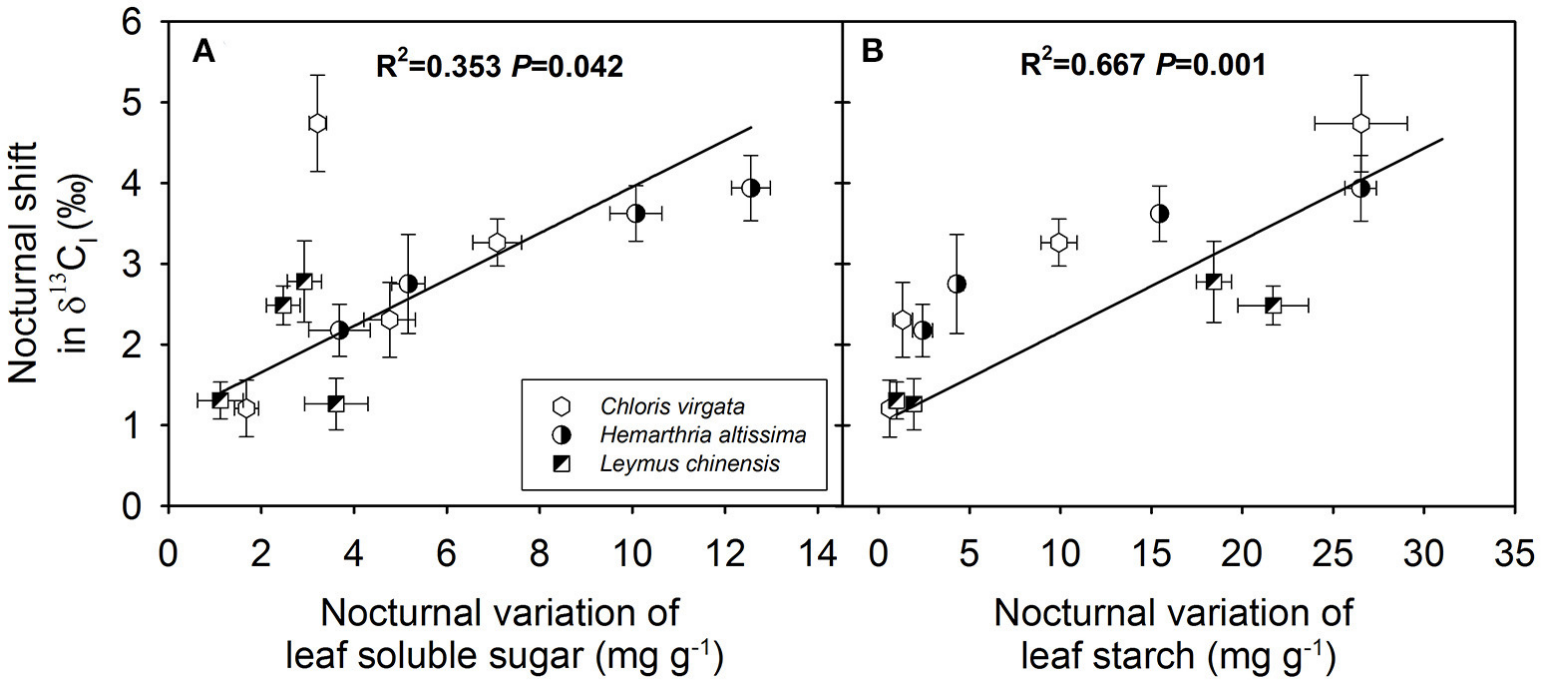
Dependence of the magnitude of the nocturnal shift in the C isotope composition of leaf dark-respired CO_2_ (δ^13^C_l_) on **(A)** nighttime variation in leaf soluble sugar (mg g^−1^) and **(B)** leaf starch (mg g^−1^) in *Chloris virgata* (hexagon), *Hemarthria altissima* (semi-filled circle) and *Leymus chinensis* (diagonal filled square). The *P* values and *R*^2^ of the linear relationship are provided.

The magnitude of the nocturnal shift in δ^13^C_l_ (maximum δ^13^C_l_ value – minimum δ^13^C_l_ value) was positively correlated with the diurnal average *A* (Figure 6A) and negatively correlated with the diurnal average *D*_l_ (Figure 6D) and diurnal average C_i_/C_a_ ratio (Figure 6E) across the studied C_3_ and C_4_ grasses. However, no significant correlations were observed between the magnitude of the nocturnal shift in δ^13^C_l_ and the nighttime average *R* (Figure 6B) or diurnal average *g*_s_ (Figure 6C). We also detected positive correlations between the magnitude of the nocturnal shift in δ^13^C_l_ and the nighttime variations in the contents of leaf soluble sugars (Figure 7A) and leaf starch (Figure 7B).

## Discussion

### Nighttime variations in the δ^13^C of leaf dark-respired CO_2_

Large nighttime variations in the δ^13^C of leaf dark-respired CO_2_ (δ^13^C_l_) were observed in both C_3_ (0.5–4.2‰ in *L. chinensis*) and C_4_ (0.5–5.9‰ in *C. virgata* and 1.3–4.9‰ in *H. altissima*) grasses, which may have resulted from changes in the carbon isotope signature of the primary respiratory substrates or shifts among substrates differing in ^13^C content (Ghashghaie et al., 2001; Sun et al., 2009). Daytime fluctuations in key environmental conditions (Brugnoli et al., 1988; Farquhar et al., 1989) and the rules of synthesis and remobilization of transitory starch (Zeeman et al., 2007) are likely to alter carbon isotope signature of nighttime respiratory substrates. The detected a strong dependence of δ^13^C_l_ on δ^13^C_soluble_ _sugar_ (Figures 5A,D,G) highlights the importance of the controlling effects of changes in the carbon isotope signature of respiratory substrates on short-term variation in the δ^13^C_l_.

Lipids are an important form of stored energy for plants to address stress conditions. In general, lipids are depleted in ^13^C more than the primary photosynthetic products (carbohydrates; Figure 2; Deniro and Epstein, 1977; Melzer and Schmidt, 1987). In a prolonged darkness study, δ^13^C_l_ in *P. vulgaris* decreased by 9‰ after 5 days of dark treatment (Tcherkez et al., 2003). This profound shift in δ^13^C_l_ was attributed primarily to the switching of respiratory substrates from soluble sugars to lipids. For the present study, we observed a strong correlation between δ^13^C_l_ and δ^13^C_lipid_ in the C_3_ grass *L. chinensis* (Figure 5I), but not in the C_4_ grasses (Figures 5C,F). These observations suggest that the observed nocturnal depletion in ^13^C in leaf dark-respired CO_2_ may have also resulted from the shifting of the respiratory substrate toward ^13^C-depleted lipids with the progression of darkness (for the C_3_ grass) and potential differences in photosynthetic type in the use of lipids to address drought stress (Xu and Zhou, 2006). In future studies, the respiratory quotient should be measured to further confirm changes in the respiratory substrate (Tcherkez et al., 2003; Gessler et al., 2009).

Moreover, nocturnal shifts in δ^13^C_l_ may be caused by the heterogeneous carbon isotope distribution of hexose molecules (Rossmann et al., 1991; Gilbert et al., 2009, 2011) and C partitioning at key metabolic branch points (Hymus et al., 2005; Priault et al., 2009). This mechanism is discussed below.

### ^13^C enrichment in leaf dark-respired CO_2_

In the present study, we detected ^13^C enrichment in leaf dark-respired CO_2_ relative to bulk biomass or primary respiratory substrates in both C_3_ and C_4_ grasses (Figure 3), which is in agreement with the results of previous studies (Duranceau et al., 1999; Ghashghaie et al., 2001; Tcherkez et al., 2003; Huxman et al., 2005; Prater et al., 2006; Barbour et al., 2007b; Werner et al., 2007; Gessler et al., 2009; Priault et al., 2009; Cui et al., 2015). The apparent ^13^C/^12^C fractionation between respiratory substrates and CO_2_ is partly attributed to the heterogeneous ^13^C distribution within hexose molecules (Rossmann et al., 1991; Duranceau et al., 1999; Ghashghaie et al., 2001; Tcherkez et al., 2003) resulting from the isotope effects of aldolase involved in the formation of fructose-1,6-bisphosphate from triose phosphates (Gleixner and Schmidt, 1997; Schmidt, 2003). Theoretically, the heterogeneous ^13^C distribution and utilization of respiratory intermediates (oxidation vs. biosynthesis) could lead to 0–4.1‰ and 0–2.3‰ ^13^C enrichment in leaf dark-respired CO_2_ relative to primary respiratory substrates in C_3_ and C_4_ species, respectively (Ghashghaie et al., 2003; Hobbie and Werner, 2004). Contrary to our expectation, large respiratory apparent isotope fractionation compared to bulk biomass and potential respiratory substrates was detected in the C_4_ grasses, which cannot be explained by the non-statistical ^13^C distribution and variation in the utilization of respiratory intermediates. Other mechanisms are likely involved in the formation of leaf dark-respired CO_2_, such as malate decarboxylation, which will generate ^13^C-enriched CO_2_ (Lehmann et al., 2015). However, photosynthetic pathway differences in the contribution of malate decarboxylation to leaf dark-respired CO_2_ flux need to be further explored.

We also observed significant correlations of the magnitude of the nocturnal shift in δ^13^C_l_ with the daily average *A* (Figure 6A) and the magnitude of nighttime variations in primary substrates (Figure 7), which suggests that the observed nighttime variations in δ^13^C_l_ may have resulted from changes in substrate-availability-associated shifts in C partitioning and subsequent apparent isotope fractionation. However, the detected nighttime changes in δ^13^C_l_ are much greater than the maximum value that can be explained by the heterogeneous ^13^C distribution theory, especially in the studied C_4_ grasses. The results suggested other mechanisms, such as, changes in the carbon isotope signature of the primary respiratory substrates, may contribute to the nighttime variation in δ^13^C_l_ in the studied C_4_ grasses. Moreover, kinetic isotope effects of respiratory decarboxylating enzymes (pyruvate dehydrogenase, isocitrate dehydrogenase and 2-oxoglutarate dehydrogenase) may increase nocturnal variation in δ^13^C_l_ (Tcherkez and Farquhar, 2005; Werner et al., 2011). In a previous study, Werner (2010) reported that the combined isotope effects of respiratory decarboxylating enzymes, relative carbon flux changes through pyruvate dehydrogenase and the TCA cycle may theoretically induce more than a 9‰ shift in δ^13^C_l_. The detected photosynthetic pathway differences in ^13^C-enrichment in leaf dark respired CO_2_ relative to biomass and respiratory substrates suggest cautions should be taken when δ^13^C of bulk biomass was used as a substitute of δ^13^C_l_.

### Drought-induced changes in the δ^13^C of leaf dark-respired CO_2_

For the studied C_3_ and C_4_ grasses, we detected significant drought impacts on the δ^13^C of leaf dark-respired CO_2_ and potential respiratory substrates (Table 1). However, the nighttime average δ^13^C_l_ on day 1 was not consistent with the variation pattern of the remaining measurement days in the two C_4_ grasses (Figure 2). These non-systematic drought effects may be attributed to the following: (1) The diurnal mean PPFD on day 1 was apparently lower than that on the remaining measurement days (Figure S1, Table S1), which may have strongly altered the photosynthetic discrimination of the studied C_4_ grasses and both the signature and magnitude of nighttime shifts in the δ^13^C of leaf dark-respired CO_2_ (Table S2). (2) On day 1, the volumetric soil water content for the C_4_ plant pots was >35% (Figure S1), which is beyond the upper limit of the optimum soil water content and may also have inhibited plant photosynthetic physiological processes. These effects can be seen from the gas exchange measurement (Figure 1).

The manipulated drought changed not only the ^13^C signature of leaf dark-respired CO_2_ but also the magnitude of nighttime variations in δ^13^C_l_. The detected strong positive correlations between δ^13^C_l_ and respiratory substrates (Figure 5) suggests that short-term variation in δ^13^C_l_ is associated with photosynthetic discrimination and the ^13^C signature of the primary respiratory substrates. Drought-induced variations in photosynthetic discrimination and the carbon isotope composition of respiratory substrates have been extensively reported (Duranceau et al., 1999; Ghashghaie et al., 2001; Williams et al., 2001). A tight correlation between δ^13^C_l_ and the δ^13^C of the respiratory substrates throughout the drought period also indicated that carbohydrate pools in the studied species turned over quickly.

The magnitude of nighttime variations in δ^13^C_l_ decreased with the intensification of the drought treatment and was strongly dependent on the diurnal average net assimilation rate (Figure 6A), diurnal average leaf-to-air vapor pressure deficit (Figure 6D) and diurnal average of the C_i_/C_a_ ratio (Figure 6E), as well as on nighttime variations in the contents of soluble sugars (Figure 7A) and starch (Figure 7B). The allocation (oxidation for energy production vs. biosynthesis of secondary compounds) of ^13^C-depleted respiratory intermediates (such as acetyl-CoA) depends on substrate availability. Changes in the availability and the magnitude of the nighttime shifts in primary respiratory substrates are the primary contributor to drought-induced variations in the range of the nighttime shift in δ^13^C_l_.

For a coexisting C_3_ and C_4_ species ecosystem, photosynthetic pathways associated with contrasting carbon isotope signatures are useful for partitioning ecosystem carbon exchange (Lai et al., 2003; Still et al., 2003; Schnyder and Lattanzi, 2005; Shimoda et al., 2009). The foliar carbon isotope composition is often used as a substitute for δ^13^C in autotrophic respiration for the separation of C_3_ and C_4_ component fluxes (Lai et al., 2003; Still et al., 2003; Schnyder and Lattanzi, 2005; Shimoda et al., 2009). However, we detected that Δ_R,biomass_ changed with plant water status and differed between the C_3_ and C_4_ plants (Table 2). More importantly, we observed that the magnitude of ^13^C enrichment in leaf dark-respired CO_2_ (relative to biomass) diminished in the C_4_ grasses, while it was enhanced in the C_3_ grass, with the intensification of the water stress (Table 2). These results suggest that the contribution of C_4_ plant-associated carbon flux is likely to be overestimated if the δ^13^C of biomass is used as a substitute for leaf dark-respired CO_2_. The studied C_3_ and C_4_ grasses demonstrated strong drought sensitivity in the carbon isotope signature and the magnitude of short term variations in leaf dark-respired CO_2_, which highlights the importance of incorporating these changes into the isotope partitioning studies.

## Conclusions

For the studied C_3_ and C_4_ grasses, δ^13^C_l_ showed a decreasing trend from 21:00 to 03:00 h. The magnitude of the nighttime shift in δ^13^C_l_ decreased with increasing drought stress. The δ^13^C_l_ values were correlated with the δ^13^C of the respiratory substrates, which suggests that the drought treatment influences δ^13^C_l_ by affecting photosynthetic discrimination. The magnitude of the nighttime shift in δ^13^C_l_ was strongly dependent on the daytime carbon assimilation rate and the range of nighttime variations in substrate availability, which indicates that changes in respiratory substrate availability may alter the allocation (oxidation for energy production vs. biosynthesis of secondary compounds) of respiratory intermediates (such as acetyl-CoA) and subsequently affect δ^13^C_l_. With the intensification of drought stress, leaf dark-respired CO_2_ in the C_4_ grasses was progressively depleted in ^13^C content, whereas leaf dark-respired CO_2_ in the C_3_ grass was enriched in ^13^C. Respiratory apparent isotope fractionation relative to biomass varied in opposite directions with the intensification of water stress between the C_3_ and C_4_ grasses. The contribution of C_4_ plant-associated carbon flux is likely to be overestimated if carbon isotope signatures are used for the partitioning of ecosystem carbon exchange and the δ^13^C of biomass is used as a substitute for leaf dark-respired CO_2_. The detected strong drought sensitivities in δ^13^C_l_ and differences in respiratory apparent isotope fractionation between the C_3_ and C_4_ grasses have marked implications for isotope partitioning studies at the ecosystem level.

## Author contributions

WS, J-YM, YL and SZ conceived and designed the experiment, SZ, HC and YX conducted the experiment, WS and SZ analyzed data. WS and SZ wrote the manuscript. All authors helped drafting the manuscript and gave essential input to the work.

### Conflict of interest statement

The authors declare that the research was conducted in the absence of any commercial or financial relationships that could be construed as a potential conflict of interest.
