# Supplementary material for: Drought Sensitivity of the Carbon Isotope Composition of Leaf Dark-Respired CO2 in C3 (Leymus chinensis) and C4 (Chloris virgata and Hemarthria altissima) Grasses in Northeast China
Source: Front Plant Sci. 2017 Dec 5;8:1996. doi: 10.3389/fpls.2017.01996 (PMC5770615; doi:10.3389/fpls.2017.01996)

Drought sensitivity of the carbon isotope composition of leaf dark-respired CO_2_ in C_3_ (*Leymus chinensis*) and C_4_ (*Chloris virgata* and *Hemarthria altissima*) grasses in Northeast China

Shangzhi Zhong^1^, Hua Chai^1^, Yueqiao Xu^1^, Yan Li^2^, Jian-Ying Ma^2^ and Wei Sun^1,*^

^1^Key Laboratory of Vegetation Ecology, Ministry of Education, Institute of Grassland Science, Northeast Normal University, Changchun, Jilin Province, P. R. China 130024

^2^State Key Laboratory of Desert and Oasis Ecology, Xinjiang Institute of Ecology and Geography, Chinese Academy of Sciences, Urumqi, P. R. China, 830011

*Corresponding author

Wei Sun

Key Laboratory for Vegetation Ecology, Ministry of Education

Institute of Grassland Science, Northeast Normal University

Changchun, Jilin Province, P. R. China 130024

Tel.: +86 431 8509 8187

E-mail address: [sunwei@](mailto:sunwei@)nenu.edu.cn

**Supplementary Table S1** Photosynthetic photon flux density (PPFD, μmol m^-2^ s^-1^) at 0600, 0900, 1200, 1500 and 1800 h on the days 1, 3, 5 and 7 of the drought treatment.

|  | **0600 h** | **0900 h** | **1200 h** | **1500 h** | **1800 h** |
| --- | --- | --- | --- | --- | --- |
| Day 1 | 321 | 445 | 412 | 332 | 129 |
| Day 3 | 453 | 1314 | 1616 | 1189 | 264 |
| Day 5 | 300 | 998 | 1486 | 1286 | 176 |
| Day 7 | 317 | 1221 | 1660 | 1157 | 242 |

**Supplementary Table S2** The photosynthetic discrimination against ^13^C (Δ_P_) at 0600, 0900, 1200, 1500 and 1800 h on the days 1, 3, 5 and 7 of the drought treatment.

| **Species** |  | **0600 h** | **0900 h** | **1200 h** | **1500 h** | **1800 h** |
| --- | --- | --- | --- | --- | --- | --- |
|  |  |  |  |  |  |  |
|  |  |  |  |  |  |  |
| ***Chloris virgata*** | Day 1 | 5.21±0.05 | 4.58±0.03 | 4.59±0.06 | 4.57±0.04 | 5.34±0.02 |
|  | Day 3 | 5.29±0.07 | 4.49±0.02 | 4.56±0.03 | 4.62±0.05 | 5.31±0.09 |
|  | Day 5 | 7.26±0.46 | 7.10±0.44 | 7.75±0.14 | 7.74±0.09 | 8.12±0.03 |
|  | Day 7 | 8.24±0.18 | 7.50±0.07 | 8.66±0.24 | 8.51±0.20 | 9.12±0.08 |
| ***Hemarthria altissima*** | Day 1 | 5.37±0.05 | 4.60±0.06 | 4.85±0.07 | 4.44±0.01 | 5.42±0.07 |
|  | Day 3 | 4.93±0.04 | 4.44±0.01 | 4.47±0.01 | 4.43±0.01 | 4.78±0.04 |
|  | Day 5 | 5.52±0.19 | 4.84±0.10 | 4.70±0.22 | 4.65±0.05 | 6.69±0.18 |
|  | Day 7 | 8.10±0.18 | 6.98±0.27 | 6.87±0.22 | 7.57±0.21 | 8.81±0.02 |
| ***Leymus chinensis*** | Day 1 | 23.71±0.23 | 20.18±0.61 | 19.60±0.77 | 17.00±0.64 | 22.06±0.52 |
|  | Day 3 | 22.67±0.91 | 20.52±0.15 | 19.88±0.29 | 18.66±0.24 | 18.00±0.98 |
|  | Day 5 | 18.91±0.85 | 20.35±1.93 | 28.7±1.31 | 23.07±1.21 | 30.14±0.36 |
|  | Day 7 | 19.68±0.64 | 24.07±0.56 | 25.40±0.39 | 24.37±0.20 | 27.09±0.26 |

Data are reported as the mean ± 1 SE (n = 5)

**Supplementary Table S3** Leaf glucose, fructose and sucrose contents at 2100, 0000 and 0300 h in *Chloris virgata* (annual C_4_), *Hemarthria altissima* (perennial C_4_) and *Leymus chinensis* (perennial C_3_) on the days 1, 3, 5 and 7 of the drought treatment.

| **Species** |  | **Glucose (mg g^-1^)** | | | **Fructose (mg g^-1^)** | | | **Sucrose (mg g^-1^)** | | |
| --- | --- | --- | --- | --- | --- | --- | --- | --- | --- | --- |
|  |  | **2100 h** | **0000 h** | **0300 h** | **2100 h** | **0000 h** | **0300 h** | **2100 h** | **0000 h** | **0300 h** |
| ***Chloris virgata*** | **Day 1** | 3.98±0.55 | 2.01±0.48 | 1.43±0.14 | 7.2±0.28 | 7.18±0.44 | 6.80±0.79 | 2.97±0.31 | 1.98±0.28 | 2.71±0.85 |
|  | **Day 3** | 4.32±0.80 | 2.60±0.66 | 3.30±0.54 | 6.02±0.66 | 7.02±0.87 | 2.87±0.17 | 7.96±0.47 | 5.02±0.60 | 5.04±0.49 |
|  | **Day 5** | 3.01±0.75 | 0.95±0.37 | 2.54±0.52 | 11.14±0.75 | 9.77±0.62 | 10.53±0.37 | 8.71±1.42 | 9.7±1.20 | 5.02±0.24 |
|  | **Day 7** | 3.95±0.43 | 3.01±0.09 | 4.37±0.72 | 8.46±0.43 | 4.46±0.06 | 6.62±0.25 | 10.27±0.95 | 12.39±1.38 | 11.37±0.32 |
| ***Hemarthria altissima*** | **Day 1** | 10.41±0.44 | 7.15±0.45 | 5.05±0.57 | 7.26±0.60 | 6.50±0.48 | 4.15±0.16 | 6.56±0.24 | 5.34±0.90 | 2.47±0.27 |
|  | **Day 3** | 5.76±0.39 | 3.35±0.18 | 4.24±0.60 | 10.12±0.52 | 6.20±0.88 | 6.84±1.08 | 8.72±0.44 | 9.64±0.90 | 3.45±0.72 |
|  | **Day 5** | 7.69±0.22 | 7.75±0.65 | 6.33±0.89 | 10.50±0.53 | 11.85±0.23 | 9.96±0.37 | 6.41±1.12 | 4.05±0.59 | 2.84±0.26 |
|  | **Day 7** | 11.50±0.63 | 9.78±0.47 | 9.08±0.70 | 10.25±0.56 | 11.43±0.99 | 11.48±0.01 | 10.64±0.33 | 8.86±0.30 | 8.22±0.22 |
| ***Leymus chinensis*** | **Day 1** | 2.82±0.53 | 3.93±0.76 | 4.05±0.54 | 8.30±0.41 | 7.20±0.60 | 6.27±0.43 | 2.83±0.58 | 2.28±0.52 | 1.15±0.19 |
|  | **Day 3** | 4.59±0.87 | 4.12±0.48 | 2.41±0.44 | 5.66±1.28 | 6.78±0.49 | 5.43±0.27 | 5.38±1.02 | 3.17±0.61 | 1.72±0.25 |
|  | **Day 5** | 3.08±0.38 | 3.14±0.22 | 1.69±0.30 | 8.21±0.46 | 8.20±1.12 | 7.81±0.54 | 3.40±0.32 | 1.36±0.77 | 1.58±1.04 |
|  | **Day 7** | 3.45±0.87 | 3.38±0.48 | 5.32±0.52 | 5.69±0.69 | 5.16±0.27 | 4.09±0.34 | 3.31±0.48 | 4.61±0.47 | 3.54±0.12 |
| Data are reported as the mean ± 1 SE (n = 5) | | |  |  |  |  |  |  |  |  |

**Supplementary Figure S1** Volumetric soil water contents (SWC-V) for the pot experiment of *Chloris virgata* (annual C_4_), *Hemarthria altissima* (perennial C_4_) and *Leymus chinensis* (perennial C_3_) on the days 1, 3, 5 and 7 of the drought treatment.

**
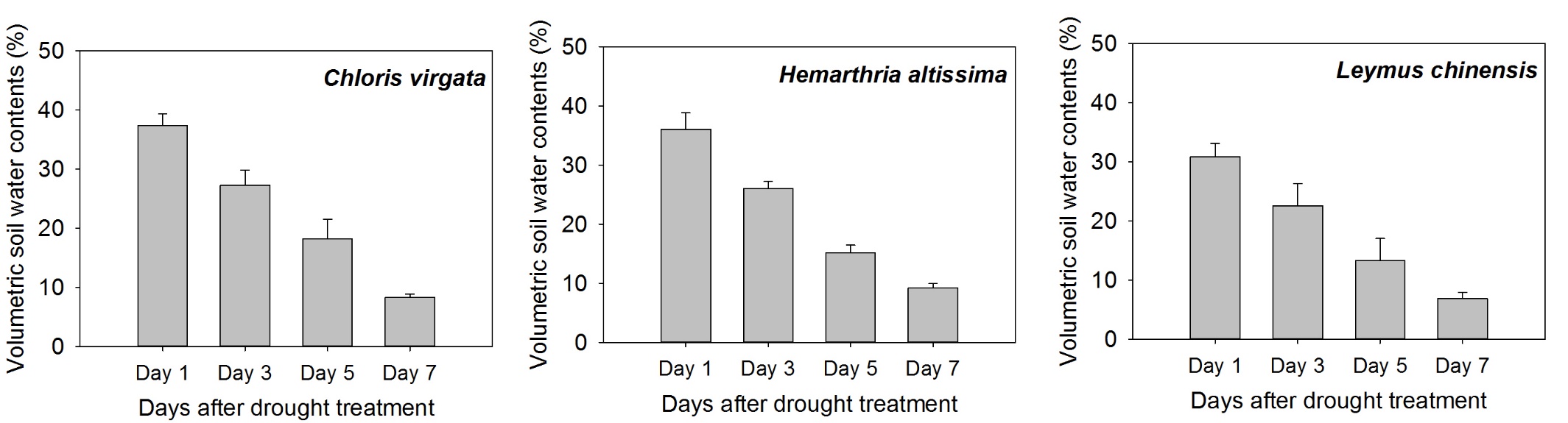
**

**Supplementary Figure S2** Meteorological data: diurnal mean air temperature (a), diurnal photosynthetic photon flux density (b), diurnal mean air relative humidity (c) and diurnal mean air saturation vapour pressure on the days 1, 3, 5 and 7 of the drought treatment.


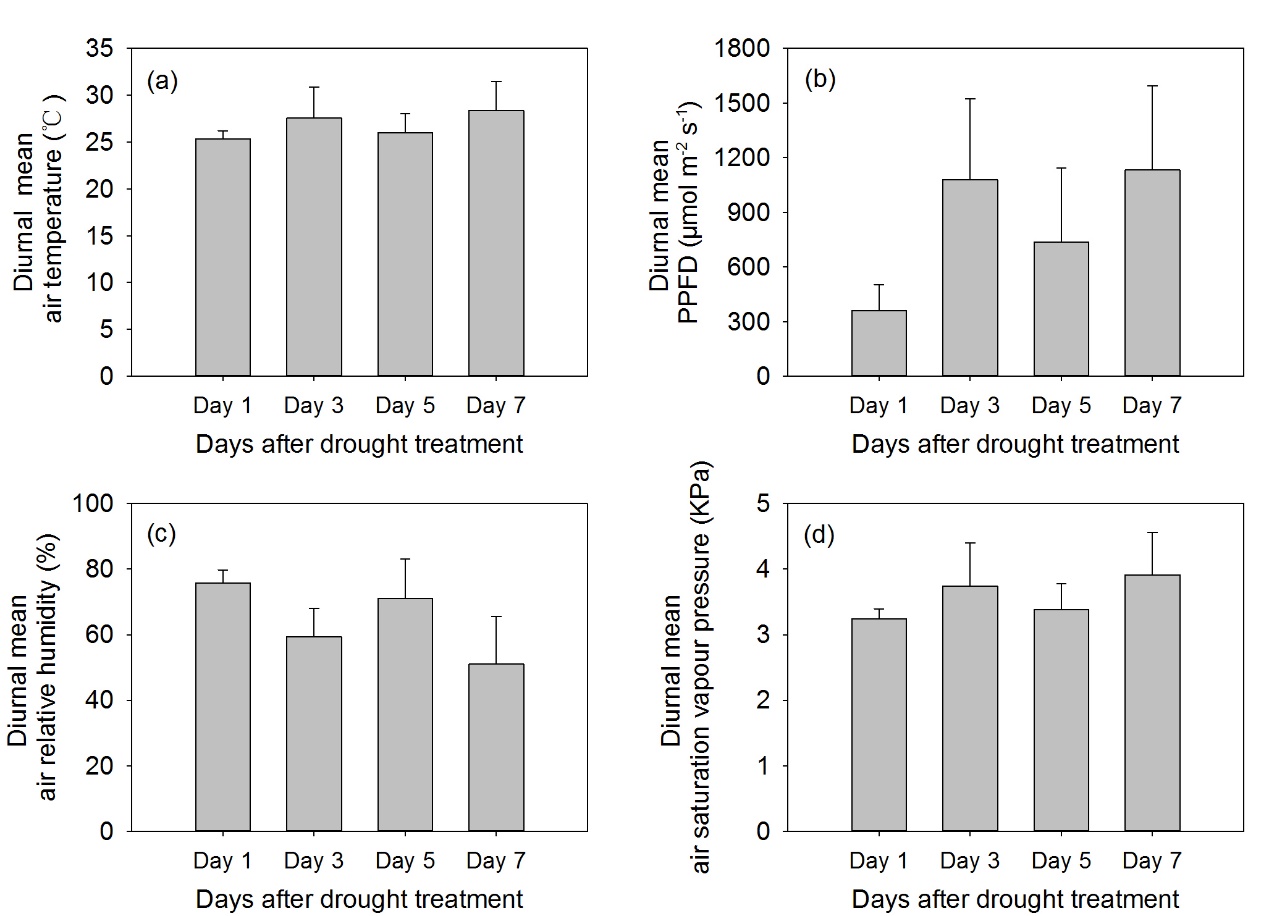

Supplement: Supplementary file 1 [file Table1.docx]
