# Supplementary material for: Drought Sensitivity of the Carbon Isotope Composition of Leaf Dark-Respired CO2 in C3 (Leymus chinensis) and C4 (Chloris virgata and Hemarthria altissima) Grasses in Northeast China
Source: Front Plant Sci. 2017 Dec 5;8:1996. doi: 10.3389/fpls.2017.01996 (PMC5770615; doi:10.3389/fpls.2017.01996)

Schematic drawing:

a. Putting syringe plunger out of the syringe and placing the leaves inside syringe (about 20 ml).

b. Connecting device interfaces:

Syringe with sample (② connect with ①)

Other syringe (no syringe plunger) with soda-lime and (③ connect with ①)

syringe needle (④ connect with ⑤)

c. Flushing the syringe barrel:

② communicate with ③ and seal ⑤, ③ connect with other syringe with soda-lime, flushed with CO_2-free air 5 times by actuating the syringe plunger

d. Collecting leaf dark-respired CO_2:

② communicate with ④ and ⑤ communicate with ⑥, CO_2 in the gas sample bag.CO_2 concentration in the gas sample bag is greater than 2000 ppm.

e. Backing to LAB and measuring CO_2 by an Isotope mass spectrometer

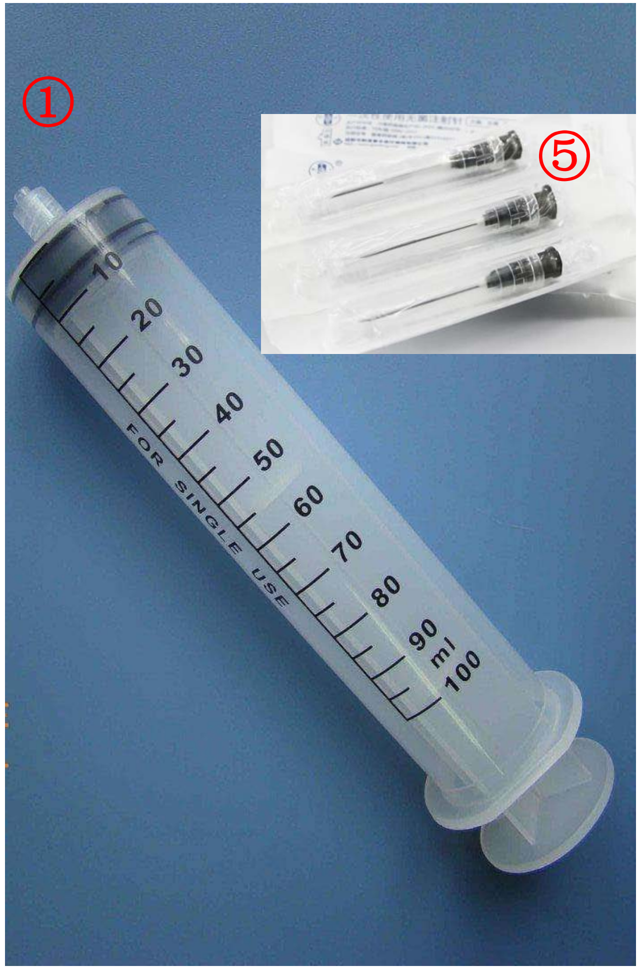

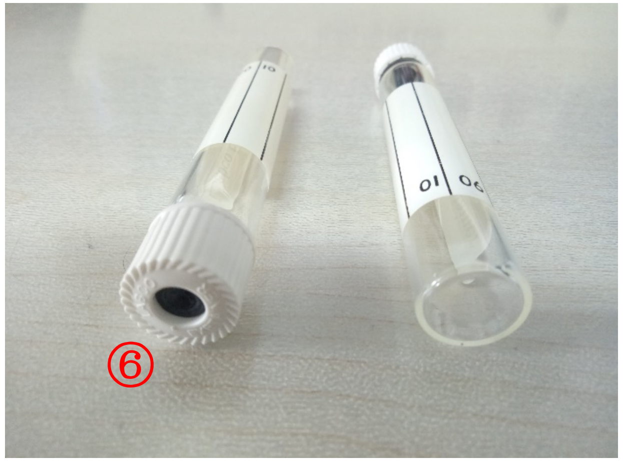

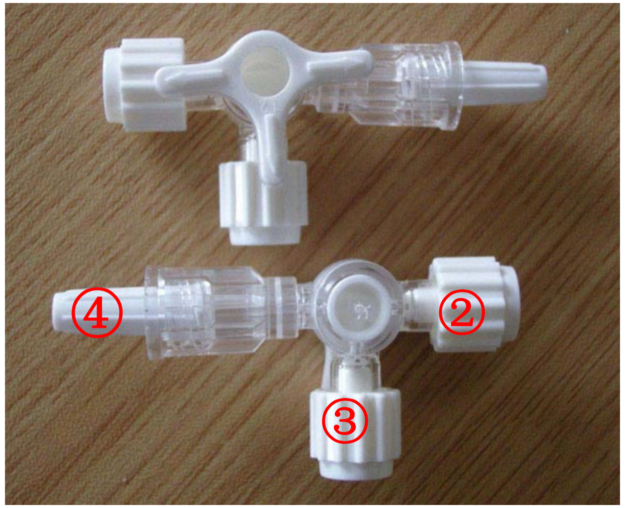

A. Air sampling equipment

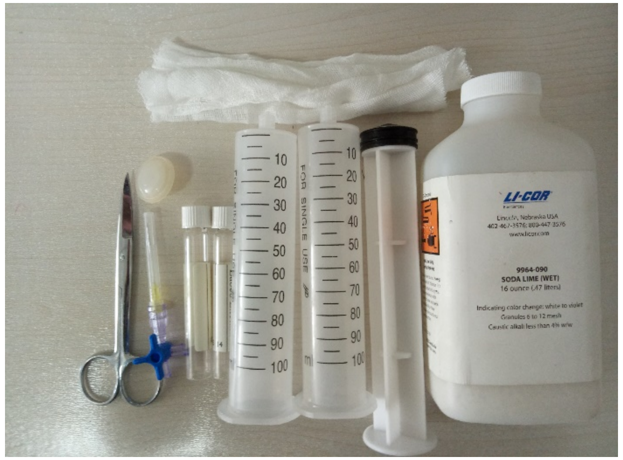

B. Putting the leaves into the syringe

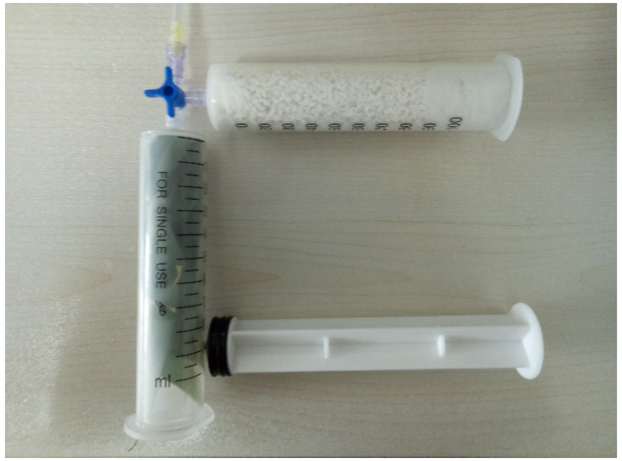

C. Flushing the syringe barrel

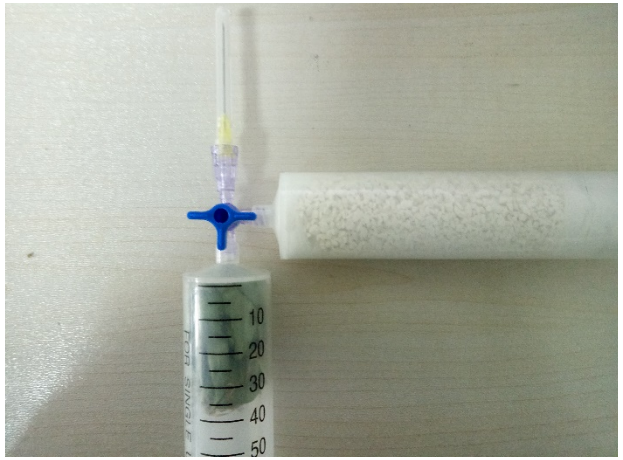

D. Accumulating CO_2 in the syringe barrel

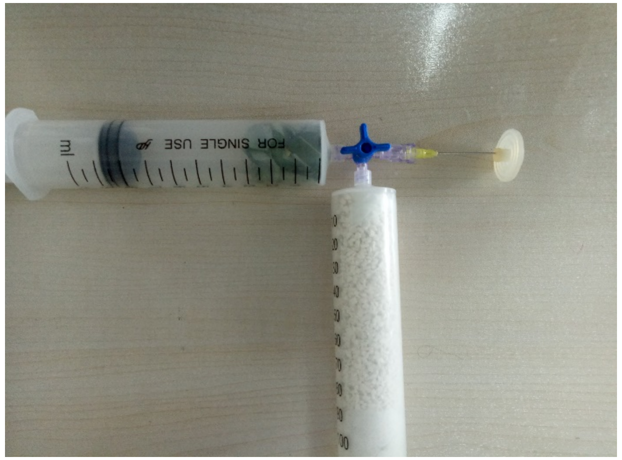

E. Collecting leaf dark-respired CO_2

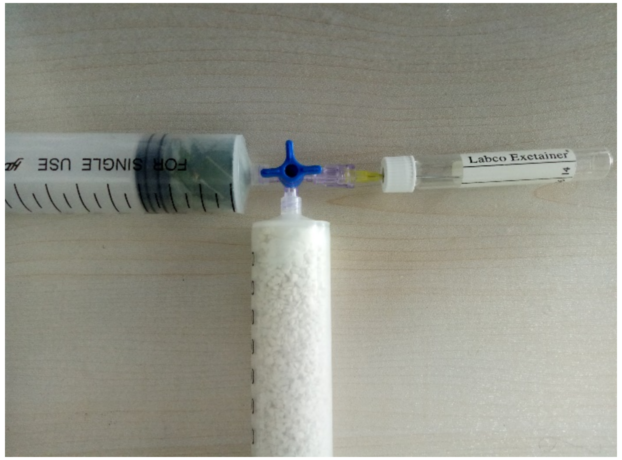

F. Backing to LAB and measuring CO_2

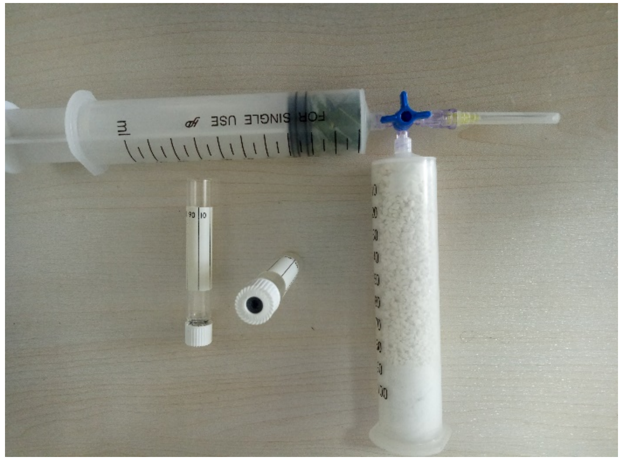

Supplement: Supplementary file 2 [file Presentation1.pdf]
